# Lactylation-drived TRIM29 induces invasive behavior and lymph node metastasis in gastric cancer via hnRNPA1-mediated Wnt/β-catenin pathway

**DOI:** 10.1038/s41419-026-08468-9

**Published:** 2026-02-13

**Authors:** Ruheng Hua, Jiawei Yu, Yuanjie Niu, Zhenwei Han, Boyang Hu, Yang Wang, Jianwei Zhu, Qingfeng Ni

**Affiliations:** Department of Gastrointestinal Surgery, Affiliated Hospital of Nantong University, Nantong, Jiangsu PR China

**Keywords:** Cancer, Gastric cancer

## Abstract

**Objective:**

Gastric cancer (GC) is a highly invasive malignancy with a propensity for lymph node metastasis. This study investigated how lactylation of TRIM29 contributes to the invasive behavior of GC and lymph node metastasis and the efficacy of chemotherapy for the disease.

**Methods:**

We examined the expression levels of TRIM29 and its lactylation status in GC tissues and cell lines using quantitative reverse-transcription polymerase chain reaction, immunohistochemistry based on tissue microarrays and western blotting. Functional transwell migration, three-dimensional invasion assay and tube formation assays were performed to assess the role of TRIM29 in GC. The interaction between TRIM29 and heteronuclear ribonucleoprotein A1(hnRNPA1) was explored by co-immunoprecipitation and mass spectrometry.

**Results:**

Expression of TRIM29 was significantly upregulated in GC tissues in comparison with adjacent non-tumor tissues. This upregulation was associated with lymph node metastasis, vascular tumors and a worse prognosis. Lactylation of TRIM29 in GC cells enhanced the migratory ability and invasiveness of these cells and lymph node metastasis. Mechanistically, TRIM29 formed a complex with hnRNPA1, which in turn activated the Wnt/β-catenin signaling pathway by stabilizing β-catenin in a ubiquitination-dependent manner. Targeting TRIM29 and lymphangiogenesis augmented the efficacy of 5-fluorouracil-based chemotherapy.

**Conclusion:**

Lactylation of TRIM29 promotes invasive behavior and lymph node metastasis in GC cells by engaging the hnRNPA1-mediated Wnt/β-catenin pathway. Targeting TRIM29 and lymphangiogenesis may be a promising therapeutic strategy for patients with advanced GC.

## Introduction

Gastric cancer (GC), ranking as the fifth most common cancer diagnosis and the fifth highest cause of cancer-related deaths worldwide, poses a substantial threat to global health. Notably, its incidence and mortality rates have shown a significant increase in the eastern Asia region, with China being particularly affected [1]. Although surgery is an effective option for some patients with GC, the characteristics of invasive growth and propensity for early metastasis deprive numerous patients of the opportunity for curative resection. The primary sites for metastasis of GC are the lymph nodes and liver, and patients with the disease have a bleak prognosis, with a reported 5-year survival rate of only 5–19% [2, 3]. Therefore, unraveling the intricate mechanisms underlying metastasis of GC is important for developing more precise and efficacious therapeutic approaches.

A variety of post-translational modification pathways have been documented to modulate the functionality of proteins and the durability of their responses to both internal and external cellular signals [4]. Ubiquitination is a key form of post-translational modification in which ubiquitin attaches to target proteins via a series of enzymatic processes, which facilitates the degradation of proteins and upholds the balance of intracellular proteins [5]. Among those, ubiquitin E3 ligase is pivotal in identifying target proteins and sustaining the ubiquitin pathway. Its malfunction can result in the aberrant build-up or over-degradation of substrates, a condition closely associated with the development and advancement of cancer [6]. Multiple studies highlight the significant function of the TRIM family, a group of E3 ubiquitin ligases comprising 75 members in humans, in orchestrating immune responses to infections and cancer therapy [7, 8]. As a member of the TRIM family, TRIM29 negatively controls antiviral immune response through STING degradation in a K48 ubiquitination-dependent manner [9]. Under endoplasmic reticulum (ER) stress, TRIM29 induces the ubiquitination-relied degradation of HMMR in hepatitis B virus (HBV)—derived hepatocellular carcinoma (HCC) progression [10]. However, the mechanism of TRIM29-induced metastasis in GC remains to be elucidated.

Epigenetic modifications, such as methylation and acetylation, are being increasingly recognized for their significant influence on a number of biological functions. The complex interactions between epigenetic alterations and metabolites are now deemed to be central in driving cancer progression and evolution [11]. An investigation by Oliver and colleagues found that metastasis of pancreatic ductal adenocarcinoma relies heavily on the oxidative arm of the pentose phosphate pathway, leading to a selective reversion of the cancer’s reprogrammed chromatin landscape [12]. Lactate has an important role in the creation of an immunosuppressive tumor microenvironment, fostering growth and metastasis of tumors by decreasing the efficacy of natural killer cells and T cells, while promoting the inhibitory activities of regulatory T cells, tumor-associated myeloid cells, tumor-associated macrophages and myeloid-derived suppressor cells [13]. Moreover, lactate-driven histone lactylation, however, is identified as a highly effective biological process in the regulation of cellular functions and pathophysiological states [14]. For example, H3K18 lactylation-driven transcriptional activation of METTL3 promotes immunosuppression of tumor-infiltrating myeloid cells in colon cancer [15]. H4K12 lactylation exacerbates microglial dysfunction in Alzheimer’s disease by activating transcription of glycolytic genes in microglia [16]. However, the correlation between lactylation of TRIM29 and the expression of this protein in GC and which type of lactylation induces expression of TRIM29, remains unclear.

In the study, we explored the role of epithelial-derived TRIM29 in the progression of GC. Our findings reveal that TRIM29, driven by H3K9 lactylation, promotes metastasis and induces lymphangiogenesis in GC. Mechanistically, TRIM29 functions independently of its ligase activity to competitively interact with the ZFP91/heteronuclear ribonucleoprotein A1 (hnRNPA1) complex, leading to a reduction of ubiquitinated hnRNPA1 and a more stable expression of hnRNPA1. We also demonstrated that 5-fluorouracil (5-FU) may be more effective in patients with limited expression of TRIM29. Therefore, targeting TRIM29 may be a potential therapeutic approach for GC.

## Materials and methods

### Clinical samples

Gastric cancer (GC) specimens were procured from patients who underwent radical gastrectomy at the Department of Gastrointestinal Surgery, the Affiliated Hospital of Nantong University. Participants did not receive adjuvant chemotherapy prior to their surgery. The collection of GC tissue samples spanned from November 2016 to October 2017. All participants provided informed consent, and the study was conducted in compliance with the Declaration of Helsinki principles and was granted approval by the Ethics Committee of the Affiliated Hospital of Nantong University under the reference number (2014-L103).

### Cell lines and culture

A HEK 293T cell line, four GC cell lines (AGS, HGC27, MKN45, and SUN-1), and a normal human gastric epithelial cell line (GES-1) were sourced from the Shanghai Institutes for Biological Sciences(Shanghai, China). The HGC27, MKN45, and SUN-1 cell lines were grown in Roswell Park Memorial Institute 1640 (RPMI-1640) medium, while the AGS cells were maintained in Kaighn’s Modified Ham’s F-12K (F12K) medium, and the HEK293T cells were cultivated in a Dulbecco’s Modified Eagle Medium (DMEM)/F12 medium (with all media supplied by Gibco, Waltham, MA, USA). Each type of medium was supplemented with 10% fetal bovine serum(Wisent, Saint-Jean-Baptiste, QC, Canada) and 1% penicillin/streptomycin (Gibco). Human lymphatic endothelial cells (HLECs) were specifically cultured in Endothelial Cell Medium (ECM) formulated with 5% fetal bovine serum (ScienCell Research Laboratories, Carlsbad, CA, USA) under conditions of 5% CO2 and a temperature of 37 °C in a humidified incubation chamber. Mycoplasma testing was performed using a Venor® Gem qEP kit (Minerva Biolabs, Berlin, Germany) at 3-month intervals to maintain cell line integrity and purity.

### RNA-associated experiments

RNA was isolated and purified from samples using the RNA-easy extraction reagent (Vazyme, Nanjing, China) following the guidelines of the manufacturer. For the synthesis of complementary DNA (cDNA), 1 µg of the extracted RNA was utilized with the Primescript RT Reagent (Vazyme, China). Quantitative real-time polymerase chain reaction (qRT-PCR) was performed using a standard SYBR Green PCR kit (Vazyme) with the Thermal Cycler Dice Detection System. The expression data were adjusted relative to the reference gene GAPDH for normalization.

### Lentivirus and transfection

To establish stable cell lines, cells were subjected to transfection via lentiviral vectors carrying either a negative control or short hairpin RNA constructs (GenePharm, Shanghai, China) designed to target TRIM29. The transfection process included the use of polybrene (at a concentration of 5 mg/mL, Sigma-Aldrich, St Louis, MO, USA) to enhance efficiency, at a multiplicity of infection of 10. At 72 h post-transfection period, the selection of stable cell lines was initiated through treatment with puromycin (at a concentration of 10 μg/mL) for three consecutive days. Flag-tagged, Myc-tagged, and HA-tagged expression vectors and mutants were sourced from GenePharm (Shanghai, China). Small interfering RNAs and plasmids were transfected into cells using Lipofectamine 3000 (Invitrogen, Carlsbad, CA, USA) in strict accordance with the manufacturer’s protocol.

### Immunohistochemistry and immunofluorescence

Tissue sections underwent deparaffinization with xylene and dehydration through a series of ethanol concentrations. Antigen retrieval was performed using a sodium citrate solution (Servicebio, Wuhan, China) in a heating device. The slides were treated with a blocking solution consisting of 10% donkey serum in 1% BSA-PBS for the duration of 1 h at ambient temperature, then exposed to the primary antibody at 4 °C overnight. After that, the slides were incubated with the appropriate secondary antibody at room temperature for 2 h. The immunohistochemical visualization was achieved with a 3,3′-diaminobenzidine (DAB) solution. The staining score was determined by multiplying the intensity score (on a scale of 0 to 4, with 0 indicating no staining and 4 indicating the strongest staining) and the percentage of stained area (scored as 0 for 0–10%, 1 for 11–25%, 2 for 26–50%, 3 for 51–75%, and 4 for 76–100% of the area). For the immunofluorescence assay, the slides or cells were incubated with Alexa-Fluor 555 or Alexa-Fluor 480 conjugated secondary antibody at room temperature for 2 h. Subsequently, the nuclei were stained with 4’,6-diamidino-2-phenylindole (DAPI). The fluorescence was examined using a laser confocal microscope (ZEISS, Germany).

### GST pull-down assays

Proteins GST-hnRNPA1 and FLAG-tagged TRIM29, encoded by the bacterial expression vector pGEX-4T-1, were produced in the Escherichia coli BL21 strain. Expression was induced using 0.4 mM isopropyl β-D-1-thiogalactopyranoside at a reduced temperature of 16 °C. The proteins were then isolated using GST affinity beads (Sigma-Aldrich, USA) and Ni2 + -NTA affinity column (Thermo Fisher Scientific, USA) following the supplier’s purification procedure.

In the GST pull-down assay, 50 μg of GST-hnRNPA1 or GST proteins bound to glutathione-Sepharose 4B beads (provided by GE Healthcare) were mixed with 20 μg of purified Flag-tagged TRIM29 and incubated for 2 h at 4 °C. Post-incubation, the protein complexes were extensively washed a minimum of four times with the GST-binding buffer. The bound proteins were eluted by heating in SDS-PAGE sample buffer and then analyzed by immunoblotting (IB) using specific antibodies.

### Co-Immunoprecipitation (Co-IP)

5 × 10^6^ cells were treated with 1 mL of immunoprecipitation (IP) lysis buffer while kept on ice for a duration of 30 minutes. After sonication and centrifugation, the supernatant was first mixed with specific primary antibodies for 4 h, and then with the Protein A/G-agarose beads (Santa Cruz Biotechnology, USA) to allow for rotating at 4 °C overnight to facilitate binding. Following this incubation, the proteins were extracted by heating the samples in an SDS-PAGE loading buffer.

### Immunoprecipitation/mass spectrometry (IP/MS)

For mass spectrometry (MS) analysis, the proteins that were immunoprecipitated were resolved using SDS-polyacrylamide gel electrophoresis, followed by visualization through silver staining. The stained protein bands were then prepared and subjected to mass spectrometry (MS) analysis for further characterization.

### Western blotting

Proteins were harvested from GC tissues and cell samples using 1× RIPA buffer (Beyotime, Shanghai, China). The proteins were resolved on a 10% or 12.5% SDS-polyacrylamide gel electrophoresis (SDS-PAGE) gel (Beyotime) and subsequently transferred onto a polyvinylidene fluoride (PVDF) membrane (Roche, Shanghai, China) which were pre-treated with a blocking buffer (Beyotime) for 20 min to prevent non-specific binding, then exposed to primary antibodies in a dilution buffer at 4 °C overnight. After incubation with secondary antibodies, the detection of protein bands was accomplished by an enhanced chemiluminescence (ECL) detection kit (EMD Millipore, Billerica, MA, USA) in conjunction with a chemiluminescent gel imaging system provided by ChemiDoc Imaging System (Bio-Rad).

### ChIP-qRT-PCR

Chromatin immunoprecipitation (ChIP) was performed using an antibody specific for the histone modification H3K9la (PTM BIO, PTM-1419RM) in strict adherence with the guidelines provided with the ChIP Assay Kit (Cell Signaling Technology,Danvers, MA, USA). The degree of enrichment of the precipitated DNA was measured by qRT-PCR and was expressed relative to the total amount of chromatin present at the start of the assay, reported as a percentage of the input. The forward primer sequence was 5′-TTTCTCCTCACCGCCTGTTC-3′, and the reverse primer sequence was 3′-TGAGGTACGGAGGGAGAAGG-5′.

### Migration assay

A total of 1.5 × 10^4^ GC cells and human lymphatic endothelial cells were plated into the upper compartment of transwell membrane filters with 8-µm pores (Corning Inc., Corning, NY, USA). The lower well was filled with medium supplemented with 10% fetal bovine serum to serve as a chemoattractant for migration. The cells were incubated at 37 °C in an environment enriched with 5% CO2 for 48 h. Following incubation, the upper surface of the membrane was cleaned, and the cells on the membrane were fixed with methanol for 10 min. The cells that had migrated through to the underside of the membrane were stained using crystal violet for 20 min.

### Three-dimensional invasion/sprouting assay

GC and human lymphatic endothelial cells were plated at a density of 2 × 10^3^ cells per well were plated into 96-well ultra-low attachment plates featuring a U-shaped well bottom (Corning, USA), and incubated for 48 h. When spheroids had formed, the cells were collected by centrifugation and then resuspended in DMEM medium that included the type I collagen (Corning, USA)with a pH value of 7–7.5. The cell spheroids were combined with type I collagen solution in a 1:1 ratio and dispensed into 96-well culture plates. They were then incubated at 37 °C for 30 min to allow the collagen to gel. When the collagen had set, 150 μL DMEM was added to each well. After a 2-day culture period, the extent of invasion from each spheroid was examined using an inverted microscope (ZEISS, Oberkochen, Germany).

### Wound healing

Cells (5 × 10^5^) were plated in 6-well culture plates (Corning, NY, USA). When confluence in the wells reached over 95%, a scratch wound was created in the middle of each well using the tip of a 200 μL pipette tip. The progression of wound closure was monitored at intervals of 0 h and 48 h and documented by photographs obtained using the inverted microscope (ZEISS, Oberkochen,Germany).

### Animal models

The Ethics Committee of Nantong University granted approval for all animal studies conducted. The nude mice utilized in these experiments were maintained in a specific pathogen-free (SPF) facility. Male mice at 6 weeks of age were selected for the establishment of all animal models and assigned to groups in a randomized manner. For the liver metastasis model, the assessment of intrahepatic metastasis involved the direct injection of 5 × 10^6^ cells suspended in 100 μL of PBS into the livers of mice, utilizing a route through the spleen’s parenchyma. To prevent tumor growth in the spleen, the spleens of the mice were surgically excised. Post a duration of 4 weeks, the mice were humanely sacrificed, and their livers were extracted for the enumeration of metastatic nodules. For the lymph node metastasis model, mice received an inoculation of 50 μL PBS containing GC cells (2.5 × 10^6^) into their footpads. The progression of lymphatic metastasis was visualized at the 4-week mark following the injection of the tumor cells. For the PDX model, tumor tissues from GC patients were kept on ice in RPMI 1640 supplemented with 10% fetal bovine serum immediately. These tissues were then trimmed into cubes of 2 × 2 × 2 mm^3^ and rinsed twice with PBS that was fortified with penicillin at a concentration of 500 U/mL and streptomycin at 500 µg/mL. Subsequently, these tissue fragments were implanted subcutaneously into the dorsal region of NSG mice at the primary (P0) generation. The entire process, from resection to transplantation, was completed within a 2-hour window. Upon reaching a size of 1.5 cm^3^ in volume, the tumor xenografts were harvested. The same preparatory steps were reiterated, and the tissue pieces were then transplanted into nude mice. Further experimental work was conducted on the second passage (P2) of mice.

### Statistical analysis

Each experimental trial was conducted three times to ensure reproducibility. The results were presented as the mean ± standard deviation (SD) and were processed using GraphPad Prism version 9.0 (San Diego, California, USA). Prior to statistical analysis, the data underwent a normality test to confirm the appropriate distribution. Inter-group comparisons were made using the Student’s *t* test linear relationships were assessed via Pearson correlation analysis, and clinical associations with clinicopathological factors were examined using the Chi-square test. Survival data were analyzed employing the Kaplan-Meier method, complemented by the log-rank test. A *P*-value of less than 0.05 was set as the threshold for statistical significance.

## Results

### TRIM29 is aberrantly expressed in GC and results in a poor prognosis

**Fig. 1:**
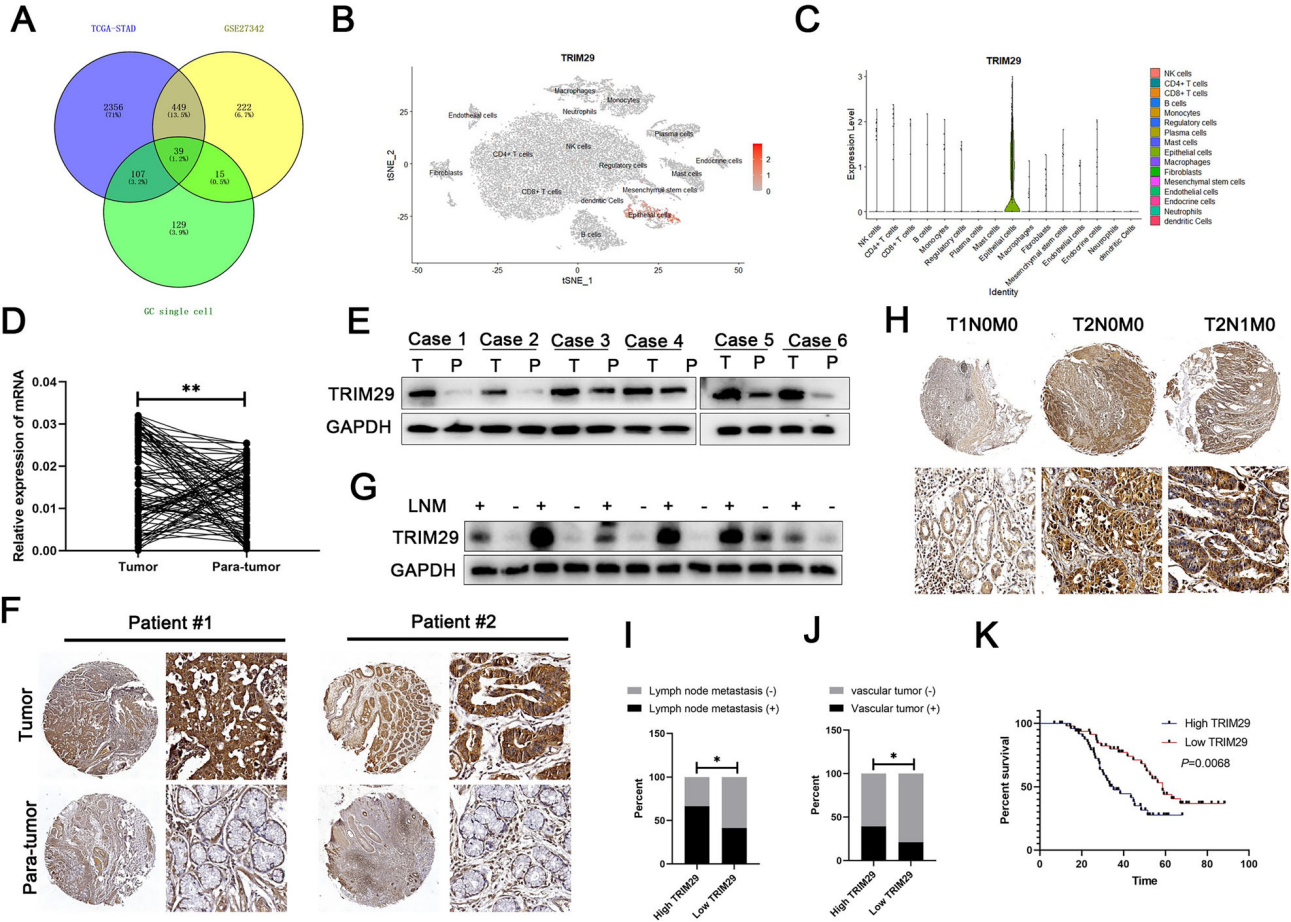
TRIM29 is aberrantly increased in GC and is an excellent prognostic biomarker for GC. **A** The genes with the most significant differential expression were identified from the TCGA database, GEO database, and single-cell database. **B** t-SNE visualization depicting the distinct cell populations and TRIM29 expression distribution. **C** A violin plot depicting the expression levels of TRIM29 across different subsets within a variety of tissue samples. Examining the levels of TRIM29 mRNA expression in GC specimens based on **D** qRT-PCR, **E** immunoblotting analysis and **F** immumohistochemistry (IHC) examination. **G** Immunoblotting analysis and **H** IHC were performed to detect the levels of TRIM29 in GC patients with lymph node metastasis or not. The influence of TRIM29 on **I** vascular tumor and **J** lymph node metastasis in GC was evaluated using parameters derived from TMA analysis. **K** Kaplan–Meier survival curves were generated to assess overall survival (OS) in GC patients with high and low TRIM29. *P < 0.05, **P < 0.01, ***P < 0.001.

Data from The Cancer Genome Atlas database and the GSE27342 and single-cell sequencing data for GC (dataset GSE163558 and GSE184198) were analyzed to identify genes associated with the progression of GC (Fig. 1A). Differential expression analysis, 39 genes were screened out for further exploration. We subsequently focused on TRIM29 which was specifically expressed in epithelial subgroup (Fig. 1B, C), and was aberrantly expressed in multiple cancers, including GC (Fig. S1A). In addition, high TRIM29 in GC was correlated with worse prognosis (Fig. S1B).

To substantiate the increased expression of TRIM29 in GC, a collection of 100 matched pairs of GC tissues and their adjacent non-cancerous tissues was examined. Obviously, GC tissues exhibited significantly higher mRNA levels of TRIM29 when compared to the adjacent normal tissues, as illustrated in (Fig. 1D). Upregulation of TRIM29 was confirmed further by western blotting (Fig. 1E) and tissue microarray (TMA) analysis, which revealed an enhanced immunohistochemical (IHC) staining score for TRIM29 in the GC tissues (Fig. 1F). Consistently, the detection in GC cell lines produced the same results, namely, expression of TRIM29 was significantly higher in AGS and HGC27 cells than in MKN45 cells (Fig. S1C, D). Evaluations of clinicopathological features combined with statistical analysis based on tissue microarray data suggested that a higher TRIM29 level was linked to the presence of vascular invasion and tumor node metastasis (TNM) classification (Fig. 1G–J) (Table 1). In addition, the Kaplan-Meier survival analysis revealed that overall survival was significantly poorer in patients with GC who had high TRIM29 levels than in those with lower TRIM29 levels (*P* = 0.0068, Fig. 1K). This finding suggested that expression of TRIM29 could be a critical indicator of the prognosis.

**Table 1:** Relationship between TRIM29 expression and clinico-pathological features in GC patients (*n* = 100).

Characteristics	TRIM29		Total	*P*
	Low expression (*n* = 50)	High expression (*n* = 50)		
**Age**				
<60	25	22	47	0.548
≥60	25	28	53	
Gender				
**Male**	27	25	52	0.689
Female	23	25	48	
**Tumor size**				
<3 cm	31	23	54	0.108
≥3 cm	19	27	46	
**Tumor site**				
Proximal	30	27	57	0.545
Non-proximal	20	23	43	
**Lymph node metastasis**				
N0	28	17	45	0.027*
N1-N3	22	33	55	
**TNM stage**				
Ⅰ-Ⅱ	26	16	42	0.043*
Ⅲ	24	34	58	
**Vascular invasion**				
Negative	39	29	68	0.032*
Positive	11	21	32	

### TRIM29 promotes GC invasive behavior and induces lymph node metastasis

In view of the correlation between the TRIM29 level and the progression of GC, we explored the role of TRIM29 both in vitro and in vivo. We employed specifically short hairpin RNAs to deplete the high levels of TRIM29 in AGS and HGC-27 cells, which exhibited high levels of TRIM29. Conversely, a lentiviral vector encoding TRIM29 was utilized for enhancing TRIM29 levels in MKN45 cells that demonstrated low TRIM29 expression. The efficiency of transfection was validated at both the mRNA and protein levels (Fig. S2A, B).

**Fig. 2:**
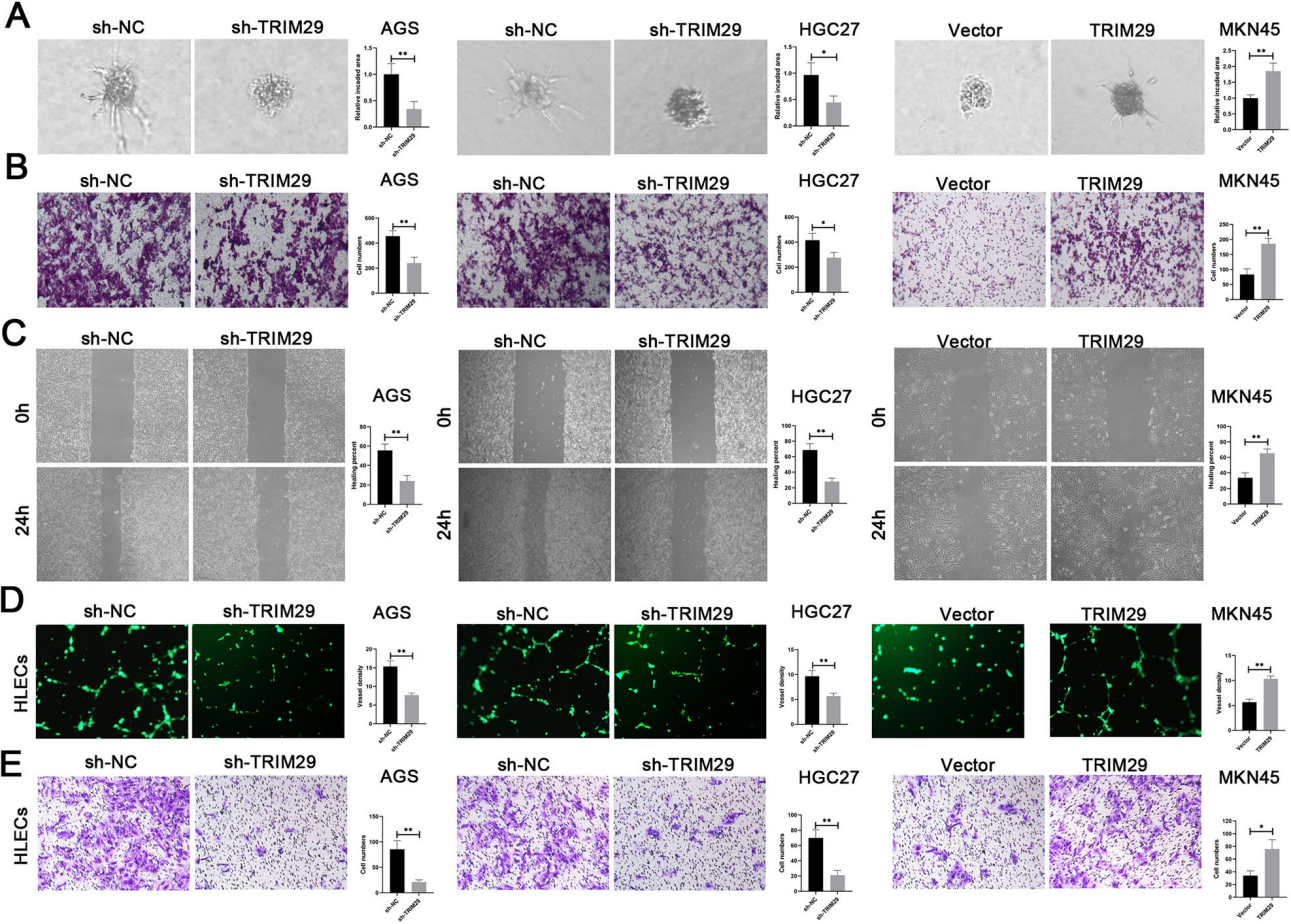
TRIM29 induces metastasis and lymphangiogenesis in GC in vitro. **A** Three-dimensional (3D) invasion and **B** transwell assays and **C** wound healing were conducted to evaluate cell invasion and migration mediated by TRIM29. **D** Tube formation assay and **E** transwell assays were performed to probe the effects of TRIM29 on lymphangiogenesis and lymphatic endothelial cell migration. *P < 0.05, **P < 0.01, ***P < 0.001.

**Fig. 3:**
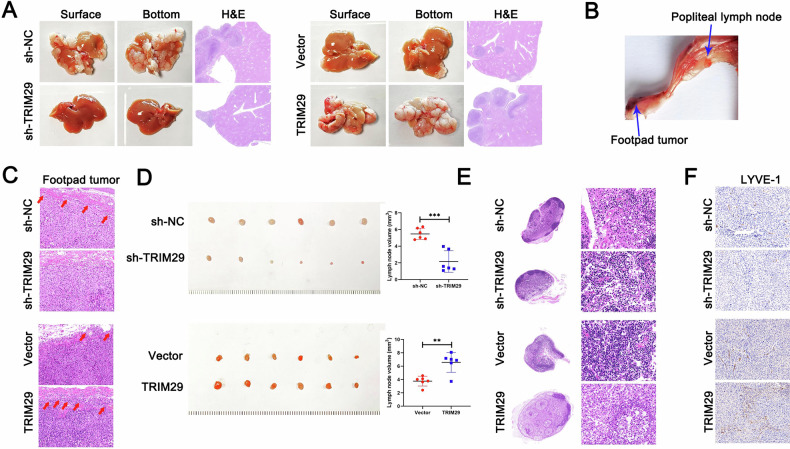
TRIM29 induces metastasis and lymphangiogenesis in GC in vivo. **A** Typical images and H&E staining depicting the liver metastasis in mice models that were inoculated with either TRIM29-silencing or TRIM29-overexpressing GC cells. **B** Illustrative images showcasing the GC popliteal lymph node (LN) metastasis model. **C** H&E staining was utilized to identify the invasive front between dermal tissue and the primary tumor located in the footpad. **D** Typical photographs of the popliteal lymph nodes (LNs) extracted from mice, along with the measured volume of LNs for each group, with a sample size of six (*n* = 6). **E** H&E staining of the tumor-filtered lymph node tissues and **F** IHC staining of the primary tumor located in the footpad were conducted to evaluate the lymphatic vessels (revealed by lymphangiogenesis marker LYVE1). *P < 0.05, **P < 0.01, ***P < 0.001.

Initially, we performed Cell Counting Kit-8 and colony formation assays to assess the impact of TRIM29 on the proliferation of GC cells. Unexpectedly, we found that TRIM29 had little effect on the proliferation of GC cells in vitro (Fig. S2C, D). However, silencing TRIM29 in the AGS and HGC-27 cell lines led to a significant decrease in their migratory ability and invasiveness. In contrast, overexpression of TRIM29 enhanced the activity of these cells (Fig. 2A–C). The similar effects of TRIM29 on the HLECs lymphangiogenesis and migration were observed (Figs. 2D and S2E). In vivo liver metastasis model showed that silencing TRIM29 displayed a decrease in both the quantity and dimensions of intrahepatic metastatic nodules when juxtaposed with the control group. In contrast, the group with TRIM29 overexpression exhibited outcomes that were contrary to those of the knockdown group (Fig. 3A). In the model of lymph node metastasis model(LNM), infiltration of GC cells into the subcutaneous tissue of the footpad was significantly reduced in mice that received the TRIM29-deplting injection. Conversely, the MKN45 cells, when engineered to overexpress TRIM29, exhibited a heightened invasive phenotype in the area of the primary tumor (Figs. 3B and S3C). The same effects were seen in the model of LMN (Figs. 3D and S3E). The in vivo results were the same for lymphangiogenesis (Fig. 3F). Combined, these results imply that TRIM29 might be a key factor in the invasive and metastatic process of GC.

### TRIM29 binds to and stabilizes hnRNPA1

**Fig. 4:**
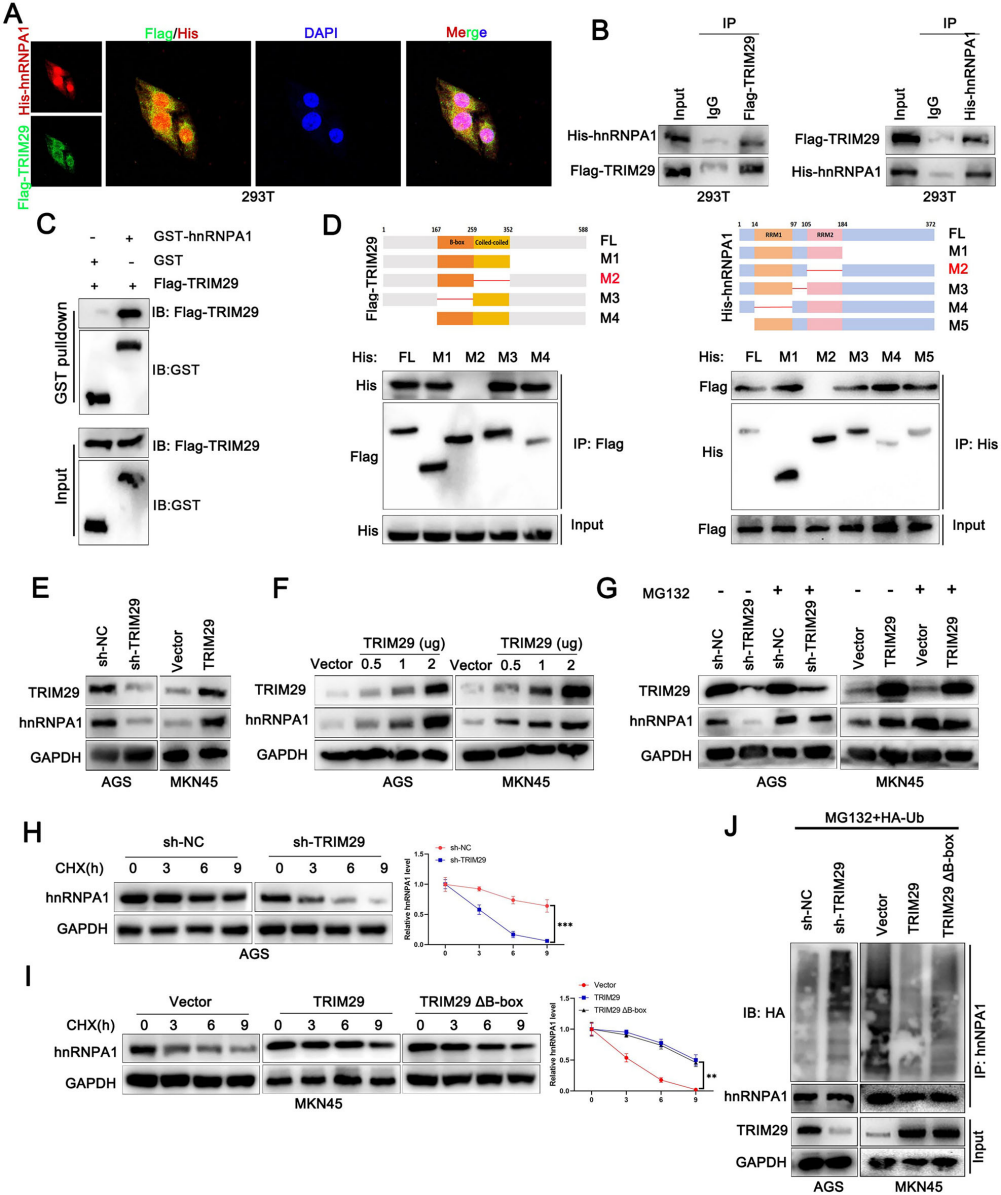
TRIM29 interacts with hnRNPA1. **A** Immunofluorescence analyses and **B** immunoprecipitation were carried out utilizing antibodies specific to Flag-TRIM29 and His-hnRNPA1 to confirm their binding. **C** Proteins Flag-TRIM29 and GST-hnRNPA1 were purified from Escherichia coli extracts. Subsequently, in vitro pull-down assays were conducted, with GST protein serving as a negative control. **D** A diagrammatic illustration of the Flag-tagged full-length (FL) TRIM29, His-tagged FL hnRNPA1, alongside a variety of their respective deletion mutants. 293T Cells were co-transfected with His-hnRNPA1 and either Flag-TRIM29 or their various deletion mutants. The cell lysates were then subjected to immunoprecipitation using anti-Flag beads, after which immunoblotting was performed using antibodies against both His and Flag tags. **E** Immunoblotting analysis was conducted to evaluate the levels of hnRNPA1 protein in cells where TRIM29 was either knocked down or overexpressed. **F** Immunoblotting was utilized to assess the expression levels of hnRNPA1 in cells that had been transfected with varying amounts of TRIM29-expressing or control vector plasmids. **G** Immunoblotting analysis was carried out to evaluate hnRNPA1 expression in cells treated with MG132 (proteasome inhibitor) or not, under TRIM29 silencing conditions or with TRIM29 overexpression. **H** The stability of hnRNPA1 proteins was assessed in cells with TRIM29 depletion, following treatment with cycloheximide (CHX) and subsequent detection at specific time points. **I** The stability of hnRNPA1 proteins was assessed in cells with TRIM29 wild type (WT) or mutant (TRIM29 Δ B-box, lacking the B-box domain) overexpression, following treatment with cycloheximide (CHX) and subsequent detection at specific time points. **J** Cells were transfected with shRNA targeting TRIM29 or a plasmid expressing TRIM29 (WT) or TRIM29 (ΔB-box) and wild-type ubiquitin (HA-Ub). Following that, cell lysates were harvested and subjected to IP using anti-hnRNPA1. The precipitated proteins were then analyzed by immunoblotting. *P < 0.05, **P < 0.01, ***P < 0.001.

In an effort to gain a comprehensive understanding of the mechanism by which TRIM29 contributes to the progression of GC, we performed immunoprecipitation followed by mass spectrometry to identify proteins that interact with TRIM29. Combining the unique peptides and coverage, heteronuclear ribonucleoprotein A1 (hnRNPA1) was identified as a potential binding partner (Figs. 4A and S3A). As a typical splicing silencer, hnRNPA1 is a member of heterogeneous nuclear ribonucleoprotein (hnRNP) family that inhibits RNA splicing [17]. Studies have shown that hnRNPA1 induces an extracellular vesicle package that promotes LNM in bladder cancer [18] and pancreatic cancer [19]. We confirmed that the hnRNPA1 level was high in both GC tissues and cell lines (Fig. S3B, D). Subsequently, Co-IP, immunofluorescence, and glutathione-S-transferase pulldown assays verified the interaction between TRIM29 and hnRNPA1(Fig. 4A–C). Molecular mapping indicated that the coiled-coil region of TRIM29 was primarily responsible for its interaction with hnRNPA1. Additionally, the segment of hnRNPA1 that binds to TRIM29 was identified in its RRM2 domain (Fig. 4D). Expression of hnRNPA1 was reduced in cells in which TRIM29 was depleted and elevated in cells with increased expression of TRIM29 (Fig. 4E). However, little change in hnRNPA1 mRNA was observed in GC cells, regardless of whether the TRIM29 level was high or low (Fig. S3E). There was no significant correlation between TRIM29 and hnRNPA1 at the mRNA level (Fig. S3F, G), suggesting that hnRNPA1 was affected by TRIM29 at the protein level rather than the mRNA level.

To investigate the TRIM29/hnRNPA1 complex further, we monitored the dynamics of the hnRNPA1 protein level in GC cells transfected with various amounts of TRIM29. The hnRNPA1 protein levels increased markedly in a TRIM29 dose-dependent manner (Fig. 4F). The ubiquitin proteasome and autophagic lysosome systems are the most common protein degradation pathways and have an important role in the maintenance of cell homeostasis and normal physiological functions. Therefore, we sought to identify which pathway was involved in the stability of TRIM29-mediated hnRNPA1.The stability of hnRNPA1 upon exposure to a changing TRIM29 level was restored by MG132, a proteasome inhibitor, but not by BafA1, an autophagy inhibitor (Figs. 4G and S3H). Furthermore, TRIM29 suppression promoted the degradation of hnRNPA1, while an increased presence of TRIM29 led to a prolonged half-life of hnRNPA1 (Figs. 4H and S4I), suggesting that TRIM29 might prevent degradation of hnRNPA1 via the ubiquitin proteasome pathway. Next, we transfected hemagglutinin-tagged ubiquitin into GC cells with depletion or overexpression of TRIM29 and investigated the variations in hnRNPA1 ubiquitination. Levels of ubiquitinated hnRNPA1 were markedly increased in the TRIM29-depleted group and decreased in the TRIM29-overexpressing group (Fig. 4J), which was in line with our hypothesis.

**Fig. 5:**
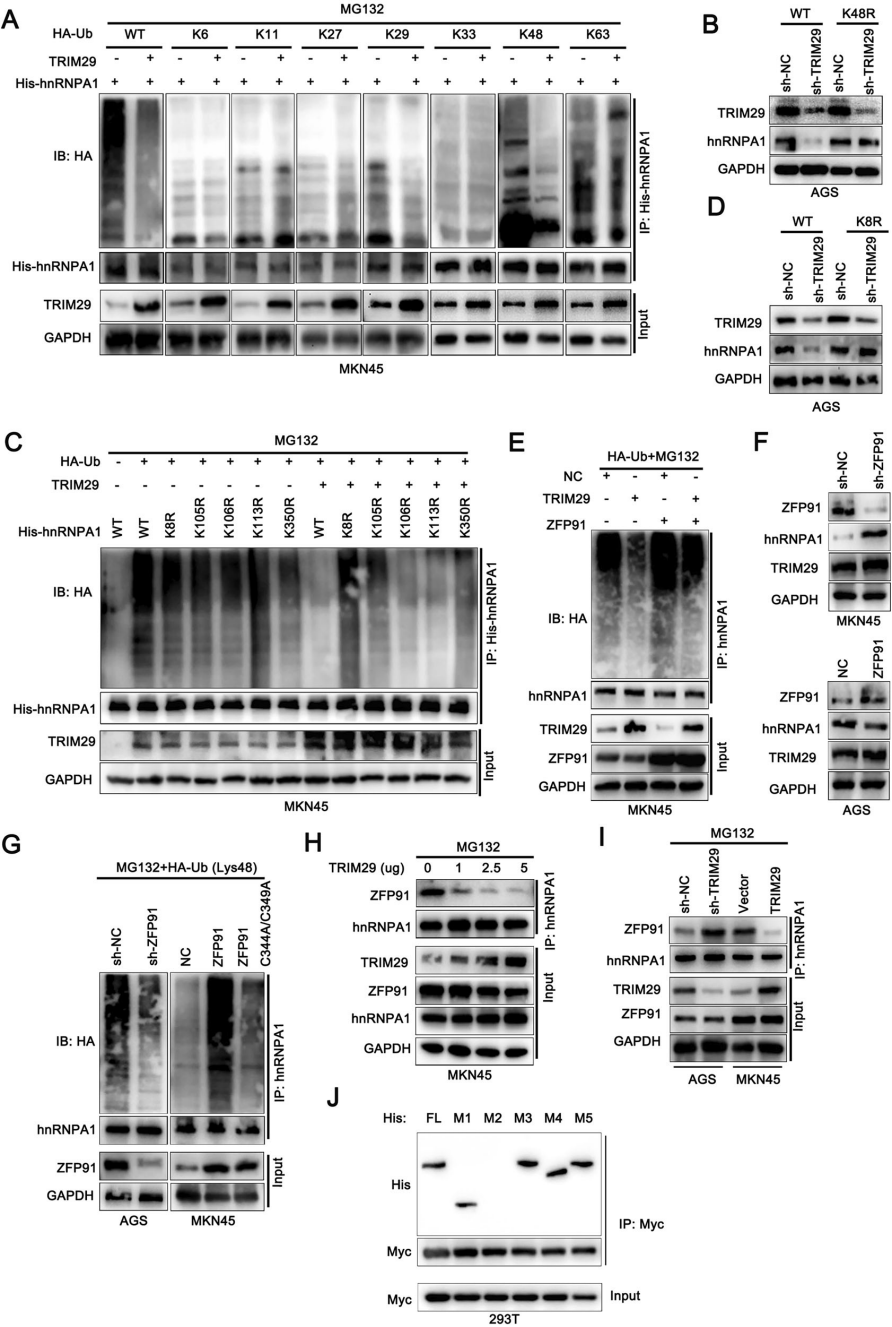
TRIM29 stabilizes hnRNPA1 by preventing ZFP91-mediated K48 ubiquitination. **A** Cells were transfected with a plasmid encoding His-hnRNPA1 and various forms of HA-tagged ubiquitin (including wild-type HA-Ub, K6-, K11-, K27-, K29-, K33-, K48-, or K63-linked Ub) along with either an empty vector or a vector expressing TRIM29 were treated with MG132 (10 μM) for 3 h. The lysates were then subjected to immunoprecipitation using an anti-His antibody and subsequent immunoblotting with an anti-HA antibody. **B** Cells transfected with either wild-type Ub or the K48R mutant ubiquitin. Then, cell lysates were examined by immunoblotting (IB) using antibodies specific to TRIM29 and hnRNPA1. **C** Cells were transfected with either the control vector plasmid or a plasmid expressing TRIM29, along with HA-Ub and either wild-type His-hnRNPA1 or its site-specific K-to-R mutant variants. Following transfection, cell lysates were prepared and subjected to IP using anti-His. The precipitated proteins were then analyzed by immunoblotting. **D** GC cells were transfected with wild-type ubiquitin (HA-Ub) and either wild-type His-hnRNPA1 or the His-hnRNPA1-K8R mutant, and cultured for 72 h in medium containing control shRNA or shRNA targeting TRIM29. After that, cell lysates were harvested and subjected to western blotting for the analysis of hnRNPA1 and TRIM29 protein levels. **E** Cells were transfected with either the control vector plasmid or a plasmid expressing TRIM29 or ZFP91, along with HA-Ub. Following transfection, cell lysates were prepared and subjected to IP using anti-hnRNPA1. The precipitated proteins were then analyzed by immunoblotting. **F** Cells were transfected with shRNA targeting ZFP91 or a plasmid expressing ZFP91. Following that, cell lysates were harvested and then analyzed by immunoblotting. **G** Cells were transfected with shRNA targeting ZFP91 or a plasmid expressing ZFP91 and its mutant ZFP91(C344A/C349A). Following that, cell lysates were harvested and subjected to IP using anti-hnRNPA1. The precipitated proteins were then analyzed by immunoblotting. **H** Cells were transfected with a plasmid expressing TRIM29 in dose relied manner. Following that, cell lysates were harvested and subjected to IP using anti-hnRNPA1. The precipitated proteins were then analyzed by immunoblotting. **I** Cells were transfected with shRNA targeting TRIM29 or a plasmid expressing TRIM29. Following that, cell lysates were harvested and subjected to IP using anti-hnRNPA1. The precipitated proteins were then analyzed by immunoblotting. **J** Cells co-expressing Myc-ZFP91 and His-hnRNPA1 or its deletion mutants were subjected to cell lysis. The precipitated proteins were analyzed by western blotting. *P < 0.05, **P < 0.01, ***P < 0.001.

We then sought to identify the specific ubiquitination event responsible for the modification of hnRNPA1. GC cells were transfected with various ubiquitin K-only constructs in which all other lysine residues were mutated to arginine. Following enrichment with anti-hnRNPA1, we detected that hnRNPA1 interacted with ubiquitin chains predominantly through K48 was altered with TRIM29 (Fig. 5A). Further investigation showed that introduction of a Lys48-resistant ubiquitin (K48R) in TRIM29-silenced cells downregulated the expression of hnRNPA1 that was normally triggered by depletion of TRIM29 (Fig. 5B). To identify the lysine sites on hnRNPA1 potentially affected by TRIM29, we created five hnRNPA1 mutants with lysine-to-arginine substitutions based on the GPS-Uber algorithm (http://gpsuber.biocuckoo.cn/wsresult.php) (Fig. S3I). Among the lysine mutants, both the K8 and K105 variants prevented TRIM29-dependent ubiquitination of hnRNPA1, with this phenomenon being more pronounced for the K8 variant (Fig. 5C). However, the reduction in expression of hnRNPA1 as a result of depletion of TRIM29 was only mitigated by the K8R mutation, implying that K8 was the detailed site responsible for hnRNPA1 K48-ubiquitination (Figs. 5D and S4A).

However, the outcome appeared to contradict the recognized role of E3 ligases in facilitating protein degradation in a K48-dependent way. Therefore, we investigated whether the greater stabilization of hnRNPA1 mediated by TRIM29 was independent of its ligase catalytic activity. The B-box domain of TRIM29 has been recognized as the catalytic core for its E3 ligase activity. We found that cells overexpressing a TRIM29 variant that lacked the B-box domain had effects similar to those of overexpressing wild-type TRIM29 (Fig. 4I, J), suggesting that the inhibition effects of TRIM29 on hnRNPA1 degradation operate independently of its ligase catalytic function. We thus speculated that TRIM29 might potentially recruit a deubiquitinase or disrupt the degradation process mediated by another E3 ligase, thereby stabilizing the target protein. However, no deubiquitinase was identified to complex with TRIM29 in our mass spectrometry data, leading us to explore alternative E3 ligases that might be responsible for the degradation of hnRNPA1. There is some evidence suggesting that ZFP91 and TRIM21 target hnRNPA1 for degradation in GC and hepatocellular carcinoma in an E3 ligase-dependent manner [20]. Both ZFP91 and TRIM21 were confirmed to interact with hnRNPA1 in GC (Fig. S4B, C). Nevertheless, it was ZFP91, rather than TRIM21, that increased the ubiquitinated hnRNPA1 level in TRIM29-overexpressing cells (Figs. 5E and S4D). We thus speculated that TRIM29 might potentially disrupt the ZFP91-driven hnRNPA1 degradation.

Subsequently, we found that depleting ZFP91 significantly increased hnRNPA1 protein levels, whereas its overexpression led to a reduction in hnRNPA1 levels. In contrast, the TRIM29 protein levels were unaffected by high or low ZFP91 levels (Fig. 5F). Furthermore, the hnRNPA1 mRNA level did not change in response to variations in the ZFP91 level, and there was no substantial correlation between the ZFP91 level and the hnRNPA1 mRNA level in GC tissues (Fig. S4E, F). Further treatment with the MG132 restored hnRNPA1 expression in cells where ZFP91 was either knocked down or overexpressed (Fig. S4G). Moreover, hnRNPA1 protein degradation was retarded by silencing ZFP91 of which overexpression decreased the hnRNPA1 ‘s half-life. However, ZFP91 mutation (ZFP91 C344A/C349A, loss of E3 ligase activity) reversed the decreased half-life of hnRNPA1 protein (Fig. S4H, I). Furthermore, the levels of K48-linked hnRNPA1 ubiquitination decreased when ZFP91 was silenced and increased following overexpression of ZFP91. However, the amount of ubiquitinated hnRNPA1 was significantly lower when a ZFP91 mutation was introduced than after transfection of wild-type ZFP91 (Fig. 5G). These results indicated that hnRNPA1 degradation in GC relied on ZFP91-mediated K48 ubiquitination. Subsequently, we detected that the binding affinity of the ZFP91/hnRNPA1 complex was decreased with TRIM29 in a dosage-dependent manner when TRIM29 was introduced (Fig. 5H). This effect occurred in cells whether TRIM29 was knocked down or overexpressed (Fig. 5I), indicating that TRIM29 may compete for binding of ZFP91 to hnRNPA1 in an E3 ligase-independent manner. Importantly, we found that ZFP91 interacted with the RRM2 domain of hnRNPA1 (Fig. 5J), coinciding with the region where the TRIM29/hnRNPA1 complex formed, as previously indicated. This finding confirmed our suspicion that TRIM29 can bind to hnRNPA1 in a competitive manner, thereby disrupting the interaction between ZFP91 and hnRNPA1.

### TRIM29 induces invasion and metastasis of GC via an hnRNPA1-mediated β-catenin pathway

Previous research has found that hnRNPA1 causes progression of cancer and induces LNM through the AKT [21], NF-κB [22, 23] and Wnt/β-catenin pathways [24]. Therefore, we sought to determine which of these pathways was affected by hnRNPA1 in TRIM29-mediated progression of GC. We found that the Wnt/β-catenin pathway, rather than the AKT or NF-κB pathway, was affected by TRIM29 in GC (Fig. S5A). TRIM29-modulated hnRNPA1 epigenetically facilitated transcription of WNT5A, thereby activating Wnt/β-catenin signaling and subsequent secretion of vascular endothelial growth factor (VEGF)-C (Fig. S5B, C), which has been reported previously [24]. We used XAV939, a β-catenin pathway inhibitor, in in vitro and in vivo models to determine whether Wnt/β-catenin signaling is essential for TRIM29-mediated progression of GC and LNM. Remarkably, increased β-catenin and VEGF-C expression levels were downregulated in TRIM29-overexpressing cells after treatment with XAV939 (Fig. S5D, E). Consistently, the rescued effects were observed in in vitro experiments including wound healing, 3D invasion and tube formation of lymphatic endothelial cells (Fig. S5F–H). We also demonstrated in an in vivo model that XAV939 significantly reversed TRIM29-mediated LNM of GC (Fig. S5I, J). Overall, these results suggested that the β-catenin pathway is essential for TRIM29-mediated invasion and LNM in GC.

We then examined whether the Wnt/β-catenin signaling pathway, modulated by TRIM29 in the progression of GC, was dependent on hnRNPA1. We transfected a plasmid expressing hnRNPA1 into cells in which TRIM29 is silenced. As predicted, overexpression of hnRNPA1 reversed the effects of TRIM29 knockdown on the β-catenin pathway and expression of VEGF-C (Fig. S6A). The findings were similar in our in vitro assays, including wound healing, three-dimensional invasion, and tube formation of lymphatic endothelial cells, and in our in vivo model of LNM (Fig. S6B–G). Collectively, the results indicated that TRIM29 induces invasive and metastatic process of GC in hnRNPA1-mediated β-catenin pathway-dependent manner.

### H3K9-drived lactylation facilitates increased transcription of TRIM29

**Fig. 6:**
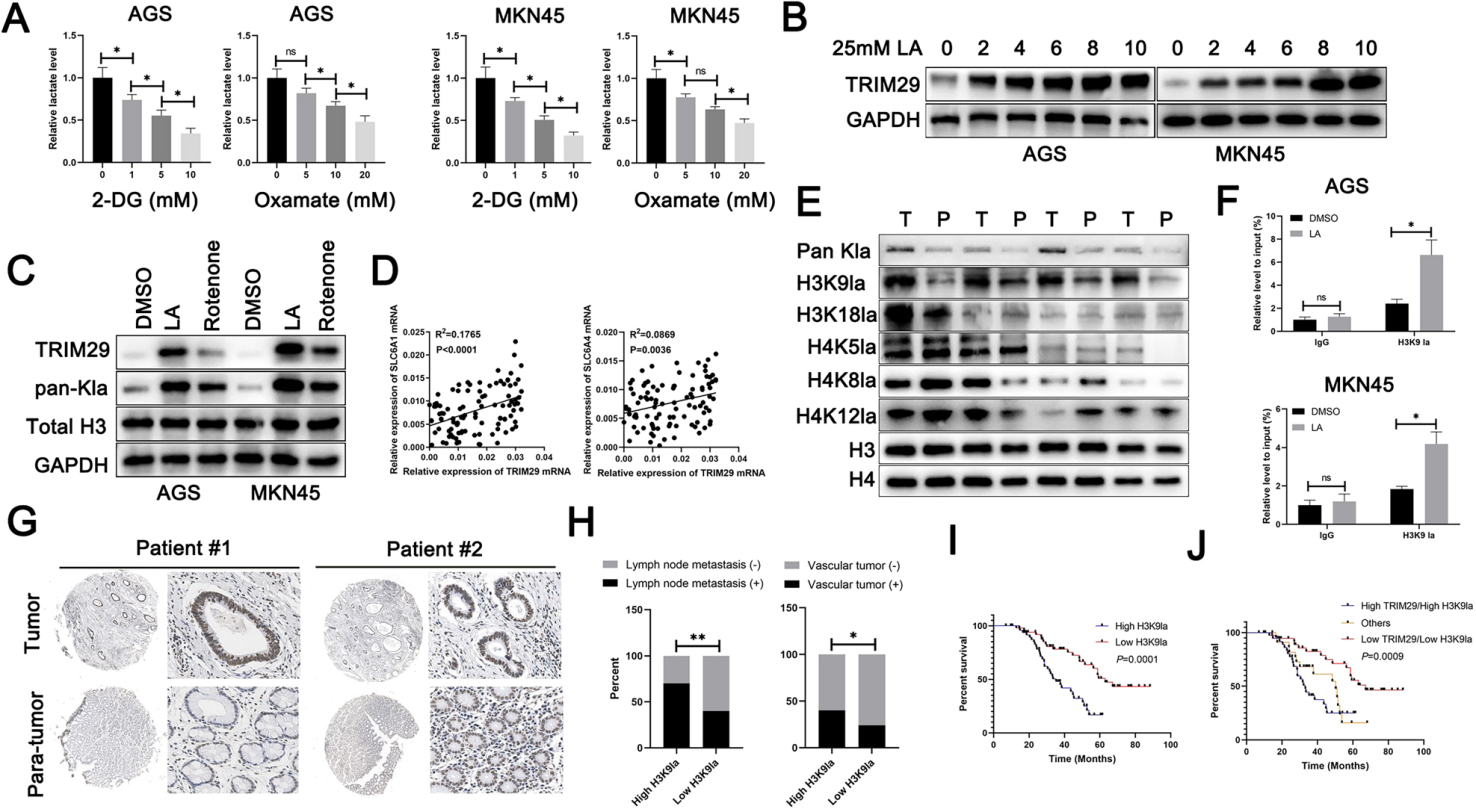
H3K9 lactylation was responsible for the increased expression of TRIM29. **A** Cells were treated with varying concentrations of 2-DG or oxamate for 24 h, the levels of intracellular lactate were subsequently quantified using a colorimetric lactate assay kit. **B** Immunoblotting was performed to analyze the expression levels of TRIM29 in whole-cell lysates derived from GC cell lines, which had been exposed to 25-mM L-lactic acid for various time periods. **C** Immunoblotting analysis was conducted to evaluate the expression of specific proteins in whole-cell lysates from GC cell lines that had been treated with either 500-nM rotenone or 25-mM L-lactic acid for a duration of 24 h. **D** Correlative analysis of TRIM29 expression levels with those of SLC6A1 and SLC6A4 was conducted utilizing RT-qPCR data. **E** Immunoblotting was used to assess the levels of global and specific-site histone lactylation in GC cells. **F** The binding of H3K9la to the TRIM29 promoter region in GC cells incubated with culture medium treated with 25-mM L-lactic acid for 24 hours was examined through ChIP-qPCR analysis. **G** IHC images demonstrating H3K9la expression in a GC tissue microarray (TMA) are presented. **H** The influence of H3K9la on vascular tumor and lymph node metastasis in GC was evaluated using parameters derived from TMA analysis. **I** Kaplan–Meier survival curves were generated to assess overall survival (OS) in GC patients with high and low H3K9la. **J** Kaplan–Meier survival analysis was conducted to evaluate the overall survival (OS) of GC patients stratified by high TRIM29/high H3K9la group, low TRIM29/low H3K9la group and other group, as determined by IHC on a tissue microarray (TMA). *P < 0.05, **P < 0.01, ***P < 0.001.

Lactate, the byproduct of aerobic glycolysis, has been reported to promote cancer development by fostering an immunosuppressive tumor microenvironment or inducing oncogene expression epigenetically through lactylation [25–27]. We speculated that lactylation could drive expression of TRIM29 in GC. As documented elsewhere [28], we used glycolysis inhibitors, including the non-metabolizable glucose analog 2-deoxy-D-glucose and oxamate, to reduce the generation of lactate and histone lactylation. We found that exposure to these agents led to a substantial dose-dependent reduction in intracellular lactate (Fig. 6A). Next, GC cells were exposed to rotenone to determine if intracellular lactate can stimulate expression of TRIM29. Rotenone disrupts complex 1 of the electron transport chain, redirecting production of ATP to glycolysis, leading to a buildup of lactate. Our findings confirmed that lactate, when accumulated endogenously, can increase expression of TRIM29, aligning with the levels of histone lysine lactylation. Subsequent L-lactic acid treatment apparently induced the increased TRIM29 expression level (Fig. 6B). To determine if intracellular lactate is capable of stimulating TRIM29 expression, GC cells were exposed to rotenone, which disrupts complex 1 of the electron transport chain, redirecting ATP production to glycolysis, consequently leading to a buildup of lactate. Our findings confirm that lactate, when accumulated endogenously, can also elevate TRIM29 expression (Fig. 6C). We also detected a significant.

Correlations of TRIM29 with SLC6A1/SLC6A4, a membrane transporter responsible for the transportation of lactic acid, were detected (Fig. 6D). We then examined a selection of distinctly recognized lactylated histone residues, including H3K18la, H3K9la, H4K5la, H4K8la, and H4K12la, for changes in their lactylation status. Notably, the consistent increase in the level of H3K9 and pan lactylation was detected (Fig. 6E). Further, ChIP-qPCR assay showed that LA treatment remarkably increased H3K9 lactylation enrichment (Fig. 6F), suggesting that H3K9 lactylation might be responsible for transcriptional activation of TRIM29. Tissue microarray analysis based on immunohistochemistry indicated that H3K9 lactylation in GC tissues was significantly higher than that in normal tissues (Fig. 6G). Moreover, survival analysis revealed that GC patients with increased H3K9 lactylation experienced worse overall survival and were more prone to lymphatic metastasis and vascular invasion (Fig. 6H, I). Together, these results demonstrated that increased H3K9 lactylation might induce expression of TRIM29 in patients with GC.

### Targeting TRIM29 or anti-lymphangiogenesis in GC may improve the potential efficacy of 5-FU-based chemotherapy

**Fig. 7:**
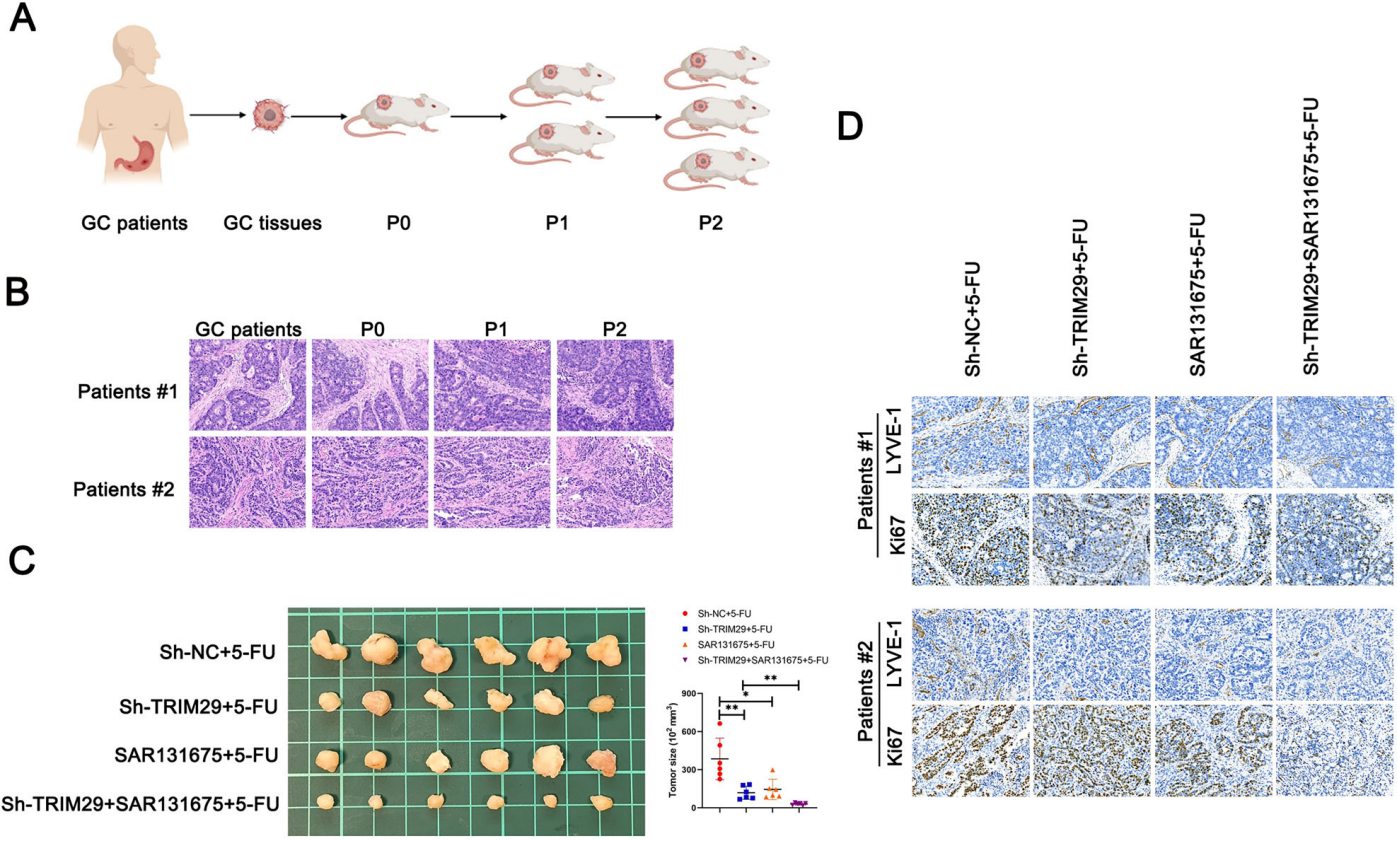
Targeting TRIM29 in GC augmented the efficacy of 5-FU. **A** A visual representation of the development of patient-derived xenograft (PDX) mouse models for GC. **B** H&E staining was conducted on GC tumor samples, as well as passages P0, P1, and P2 to verify their identical tissue origin. The antitumor effects of 5-FU, anti-lymphangiogenesis (SAR131675, a selective VEGFR-3 inhibitor) and the inhibition of TRIM29 in GC were assessed using PDX model, with (**C**) representative tumor images and size, **D** the number of lymphatic vessels and ki67 positive cells were presented. *P < 0.05, **P < 0.01, ***P < 0.001.

Suboptimal concentration of anti-neoplastic agents within tumors is a key limitation of existing cancer therapeutics, and it poses an even greater challenge in terms of the clinical application of nanomedicines [29]. In addition to the vasculature and stroma, tumor-associated lymphatic vessels might be another critical factor affecting drug accumulation in tumors. Therefore, we confirmed the clinical translation value of targeting TRIM29 and anti-lymphangiogenesis in a patient-derived xenograft model of GC (Fig. 7A, B). We found that targeting TRIM29 or inhibiting lymphangiogenesis both substantially improved the efficacy of 5-FU, with a more pronounced effect observed in the group that utilized both strategies (Fig. 7C, D). Cancer-suppressing effects were consistently observed, as revealed by Ki67 staining. These data suggested that targeting TRIM29 and inhibiting lymphangiogenesis might exert a synergistic effect that enhances the efficacy of 5-FU-based chemotherapy in GC.

## Discussion

Despite recent advances in the diagnosis and treatment of GC, many factors, including LNM, remain important in terms of the prognosis. In our study, exploration of single-cell RNA sequencing data identified that the invasive behavior of GC was regulated by TRIM29 and that lymphangiogenesis occurred via the hnRNPA1-activated Wnt/β-catenin pathway. We also confirmed that selectively targeting TRIM29 or lymphangiogenesis could improve the efficacy of 5-FU-based chemotherapy. This finding suggests that TRIM29 might serve as a prognostic biomarker and a reliable predictor of the response to chemotherapy in patients with advanced GC.

Lactic acid functions not merely as a metabolite but also as a significant epigenetic modification molecule, capable of modulating cell metabolism and immune function by influencing the lactic acidation of various histone proteins. Moreover, lactic acid can attract regulatory T cells (Tregs) via the G protein-coupled receptor 81 (GPR81) and suppress the activity of CD8 T cells. Lactic acid further dampens the anti-tumor immune response by impeding the maturation and functionality of dendritic cells (DCs) [30]. This study, for the first time, uncovers that the lactate modification of TRIM29, triggered by lactic acid, not only drives its abnormal up-regulation but also propels the progression of gastric cancer, thus shedding new light on the understanding of gastric cancer.

The Ubiquitin-Proteasome System is an intricate cellular mechanism for protein degradation that marks proteins with the ubiquitous small protein ubiquitin, enabling their targeted recognition and subsequent degradation or signaling by the 26S proteasome [31]. This process, known as ubiquitination, involves a cascade of enzymes, including E1 (ubiquitin-activating enzyme), E2 (ubiquitin-conjugating enzyme), and E3 (ubiquitin ligase), which successively attach ubiquitin to specific cellular proteins [32]. The E3 ubiquitin ligases play a pivotal role in this pathway by catalyzing the attachment of ubiquitin to the protein substrate, ensuring the specificity of the substrate for degradation [33]. The TRIM protein family constitutes a significant subgroup within the E3 ubiquitin ligase superfamily, which includes over 80 distinct proteins [34]. A member of the TRIM family, TRIM29 is involved in signal transduction, cell proliferation and differentiation, autophagy, and apoptosis [35]. TRIM29 is distinct from other members of the TRIM family owing to the absence of the RING finger domain, which confers E3 ligase activity. The B-box domain in TRIM29 performs the E3 ubiquitin ligase function, albeit with less potency than the standard RING finger domain [36]. Ubiquitin has seven lysine sites (i.e., K6, K11, K27, K33, K29, M1, K48, and K63) that enable the creation of diverse ubiquitin chain configurations, which in turn increase the complexity and regulation of ubiquitination [37]. In this study, we confirmed that TRIM29 enhances the stability of hnRNPA1 in a K48-dependent manner. However, this finding is paradoxical in terms of the typical role of the E3 ligases that promote the degradation of proteins. Therefore, we hypothesized that this process occurs independently of the ligase activity of TRIM29. We confirmed that TRIM29 competitively binds to the RRM2 domain of hnRNPA1, displacing the binding of ZFP91, another E3 ligase, thereby inhibiting degradation of hnRNPA1, which is in line with our hypothesis and provides new insights into the function of TRIM29.

Increased formation of lymphatic vessels and metastasis to lymph nodes are critical stages in the progression of cancer and are associated with an unfavorable outcome [38]. The lymphatic network undergoes significant changes during metastasis of cancer, with generation of new lymphatic vessels and restructuring of existing ones being deemed crucial for progression of metastatic disease [39]. It is well established that lymphatic vessels play a key role in the spread of metastatic GC cells. For example, Ma et al. identified reduced expression of kallistatin in GC tissues, metastatic lymph nodes, and plasma, with a lower plasma level being inversely associated with the stage of LNM. At the molecular level, kallistatin was found to suppress tumor lymphangiogenesis and lymphatic spread by reducing the expression and release of VEGF-C through modulation of the NF-κB pathway [40]. Furthermore, oxidized low-density lipoprotein in plasma, a risk factor for the development of cancer in individuals with dyslipidemia, can activate the NF-κB pathway, thereby fostering lymphangiogenesis and lymphatic dissemination in GC [41]. High expression levels of sterol oxygen-acyltransferase 1 (SOAT1) are found in more advanced GC and correlate with LNM and an unfavorable prognosis. SOAT1 is thought to promote lymphangiogenesis and LNM by regulating the expression of SREBP1 and SREBP2, which are cholesterol metabolism-related genes, without repeating the previously mentioned mechanisms [42]. A recent study has reported that irregular hypermethylation of cytosines in CpG islands results in epigenetic suppression of TRIM29 expression and activates immune response in GC, hinting that TRIM29 is a tumor suppressor [43]. In our study, however, we demonstrated that TRIM29 is a key oncogene inducing invasive behavior, lymphangiogenesis and lymph node metastasis. Similarly, in bladder cancer, TRIM29 acts as an oncogene by interacting with Keratin 14, enhancing cell migration and invasion through stabilizing focal adhesions [44]. In ovarian cancer, TRIM29 overexpression is linked to tumor aggressiveness and chemoresistance, potentially through the activation of specific signaling pathways that enhance cell survival and proliferation. In contrast, TRIM29 exhibits tumor suppressive functions in HCC. TRIM29 may inhibit tumor cell proliferation and promote apoptosis by modulating the expression of cell cycle and apoptosis-related genes [45]. Overall, TRIM29’s multifaceted roles highlight the importance of considering tumor type-specific contexts when evaluating its function and potential as a therapeutic target. Future research should focus on elucidating the precise molecular mechanisms underlying TRIM29’s actions in different cancers to fully harness its potential in cancer therapy.

While surgery is the main treatment for early GC, patients with advanced stages are typically treated with chemotherapy or immunotherapy. At present, the standard chemotherapy protocols are FOLFOX, which combines oxaliplatin, leucovorin, and 5-FU, and XELOX, which consists of oxaliplatin and capecitabine. However, there is growing concern regarding the increasing prevalence of drug resistance with the extensive use of these chemotherapeutic agents [46]. Another limitation is insufficient intratumoral drug accumulation. There is some research demonstrating that tumor-associated lymphatic vessels not only play an important role in the spread of cancer cells but also cause efflux of drugs from tumor tissue [29]. Therefore, targeting tumor-associated lymphangiogenesis might be a useful treatment strategy in patients with advanced GC. The findings of our study confirm that targeting lymphangiogenesis inhibits the growth of cancer and that a 5-FU-based regimen that targets both TRIM29 and lymphangiogenesis may be even more effective. Even though LNM is a primary route of cancer spread and is associated with an unfavorable outcome in multiple types of cancer, including GC, specific and effective clinical treatments for LNM are lacking [47] and warrant further exploration.

This study has identified that TRIM29 has an oncogenic role in GC, inducing invasive behavior and lymphangiogenesis and heralding worse survival for patients. Mechanistic exploration revealed that TRIM29, independent of the ligases' activity, activates the Wnt/β-catenin pathway and facilitates VEGFC release by inhibiting ZFP91-mediated hnRNPA1 ubiquitination. From a clinical standpoint, our patient-derived xenograft data suggest that targeting TRIM29 and lymphangiogenesis would improve the efficacy of 5-FU as monotherapy. These findings deepen our understanding of the progression of GC and could lead to the identification of novel therapeutic targets for patients with GC.

## Supplementary information

Supplemental Figure

Supplemental Material --WB original

Reproducibility checklist

## Data Availability

The datasets used and/or analysed during the current study are available from the corresponding author on reasonable request.
