# Supplementary material for: Lactylation-drived TRIM29 induces invasive behavior and lymph node metastasis in gastric cancer via hnRNPA1-mediated Wnt/β-catenin pathway
Source: Cell Death Dis. 2026 Feb 13;17(1):222. doi: 10.1038/s41419-026-08468-9 (PMC12921344; doi:10.1038/s41419-026-08468-9)
Supplement: Supplementary file 1 — Supplemental Figure [file 41419_2026_8468_MOESM1_ESM.doc]

| 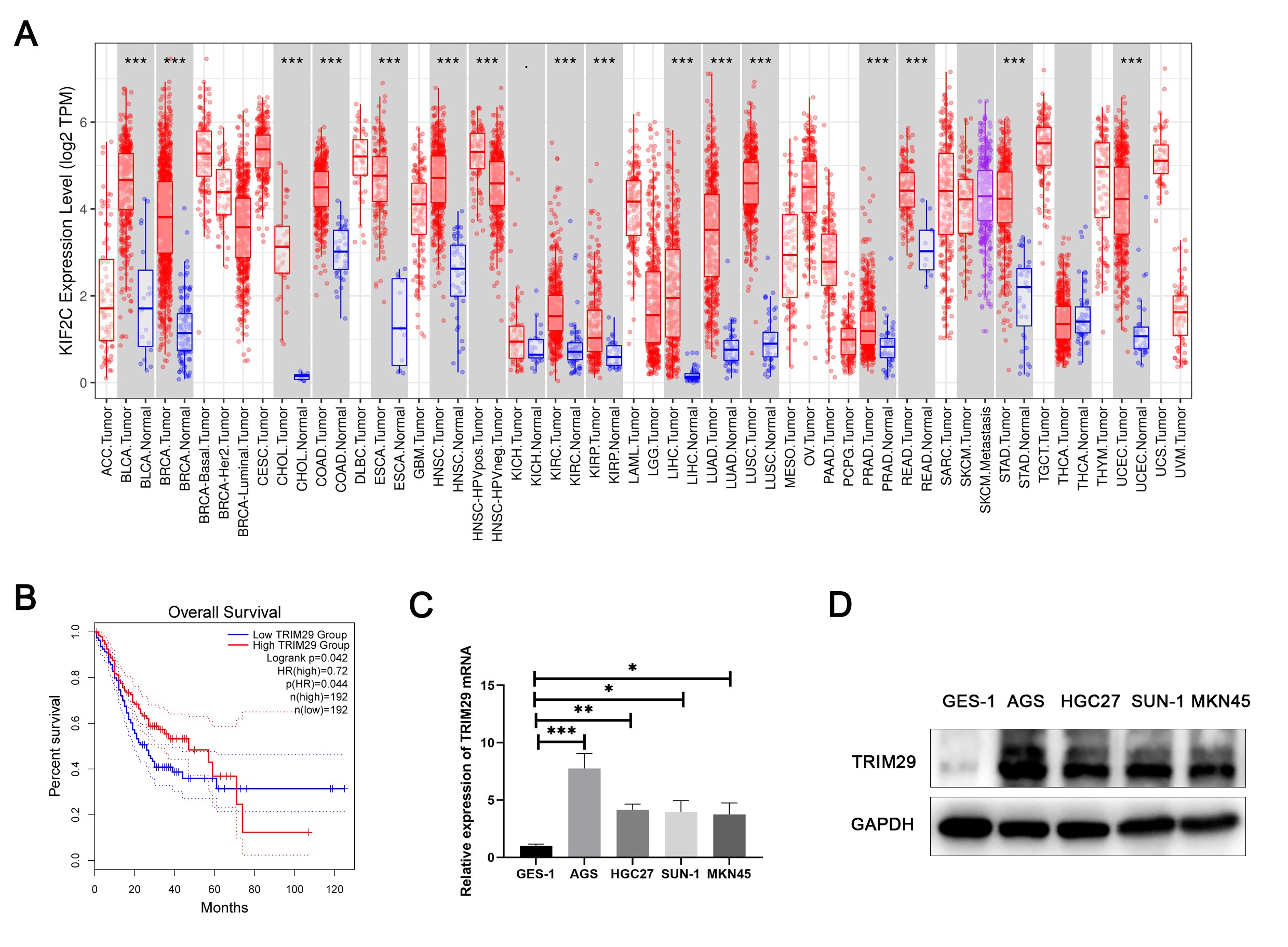 |
| --- |
| **Figure S1. TRIM29 is aberrantly expressed in multiple cancers. A,** Pan-cancer analysis of TRIM29 expression in many cancers based on online TIMER database (http://timer.cistrome.org/). **B,** The overall survival analysis of TRIM29 in GC based on TCGA database. **C,** qRT-PCR and **D,** immunoblotting analysis were utilized to detect the expression level of TRIM29 in GC cell lines, namely AGS, HGC27, MKN45, SUN-1 along with a normal human gastric epithelial cell line, GES-1.**P < 0.05, **P < 0.01, ***P < 0.001*. |

| 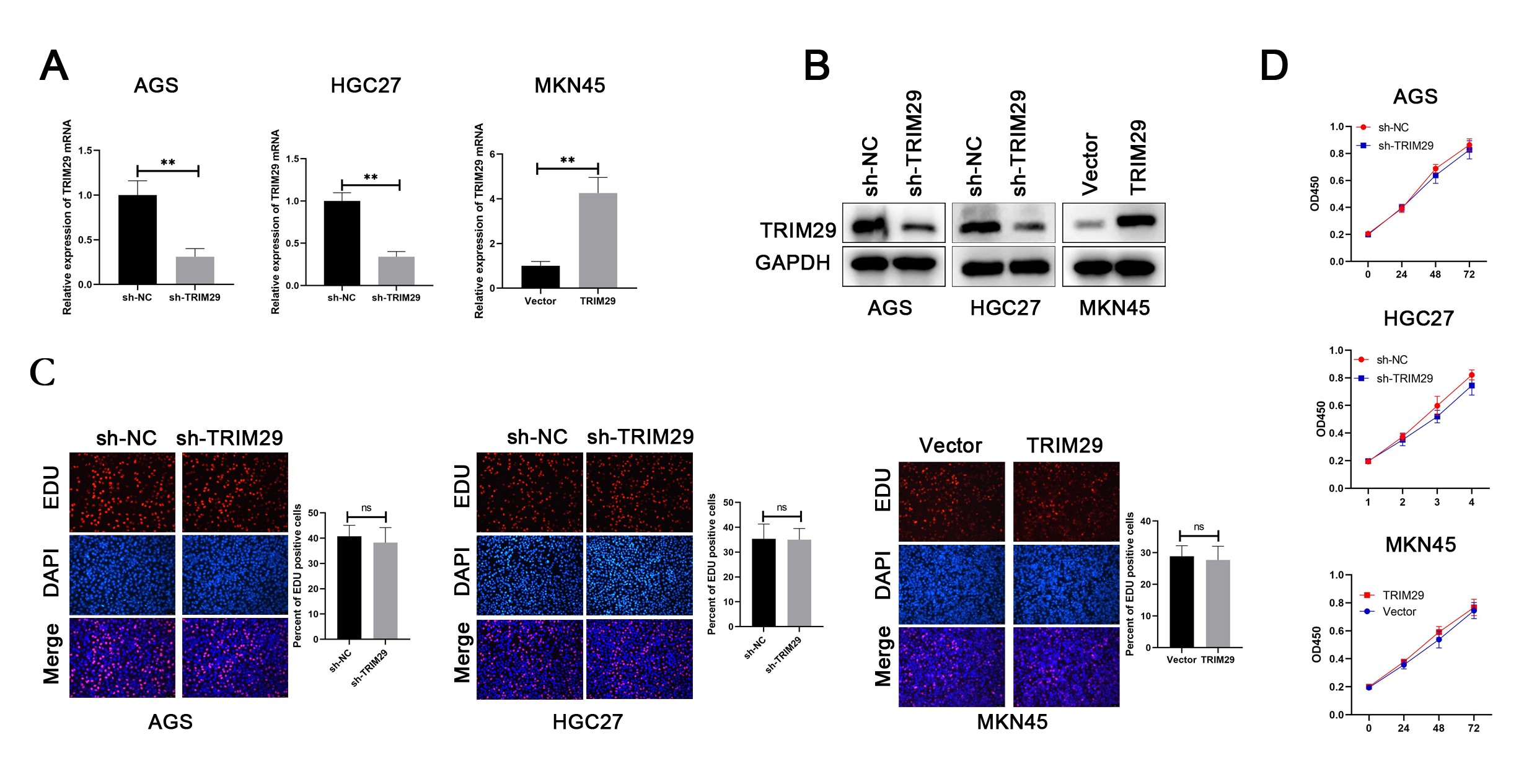 |
| --- |
| **Figure S2. GC Proliferation is not altered by TRIM29.** Transfected efficiency of TRIM29 was confirmed by **A,** qRT-PCR and **B,** immunoblotting analysis. The effects of TRIM29 on GC proliferation in vitro were detected by **C,** EDU and **D,** CCK-8 analysis. **P < 0.05, **P < 0.01, ***P < 0.001*. |

| 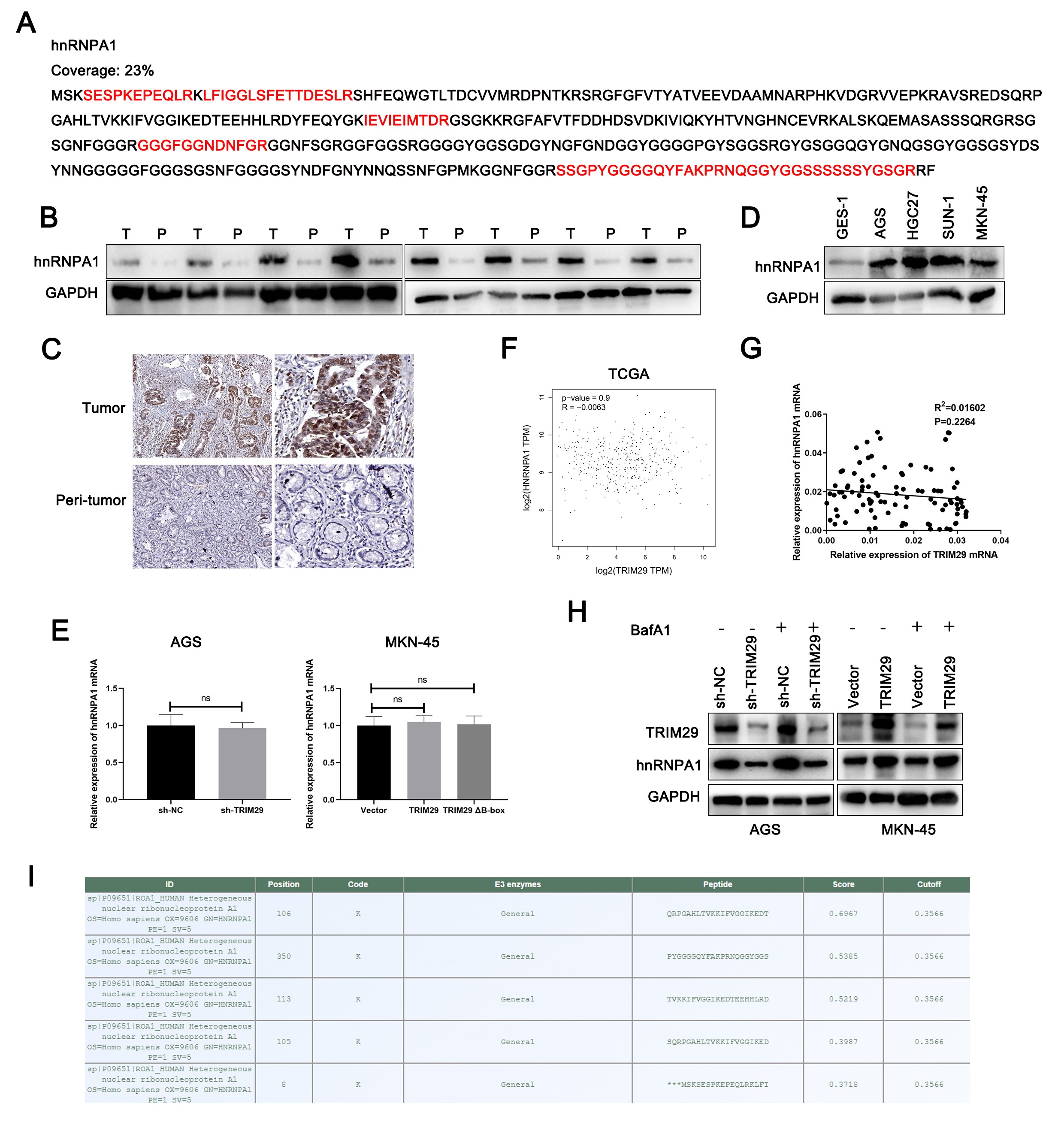 |
| --- |
| **Figure S3. hnRNPA1 is a binding partner of TRIM29 in GC.** **A,** The hnRNPA1 peptides detected in the TRIM29-enriched fractions were highlighted in red. The protein level of hnRNPA1 in GC tissues was evaluated by **B,** immunoblotting and **C,** immumohistochemistry (IHC). The protein level of hnRNPA1 in GC cell lines, namely AGS, HGC27, MKN45, SUN-1 along with a normal human gastric epithelial cell line, GES-1 was evaluated by **D,** immunoblotting. **E,** The hnRNPA1 mRNA in GC cell was explored with TRIM29 depletion or overexpression. The correlation between TRIM29 mRNA and hnRNPA1 mRNA was analyzed based on **F,** TCGA database and **G,** GC tissues from our center. **H,** Immunoblotting analysis was carried out to evaluate hnRNPA1 expression in GC cells treated with BafA1 (autophagy inhibitor) or not, under TRIM29 silencing conditions or with TRIM29 overexpression. **I,** The ubiquitinated sites of hnRNPA1 was searched and predicted according to GPS-Uber (http://gpsuber.biocuckoo.cn/wsresult.php). **P < 0.05, **P < 0.01, ***P < 0.001*. |

| 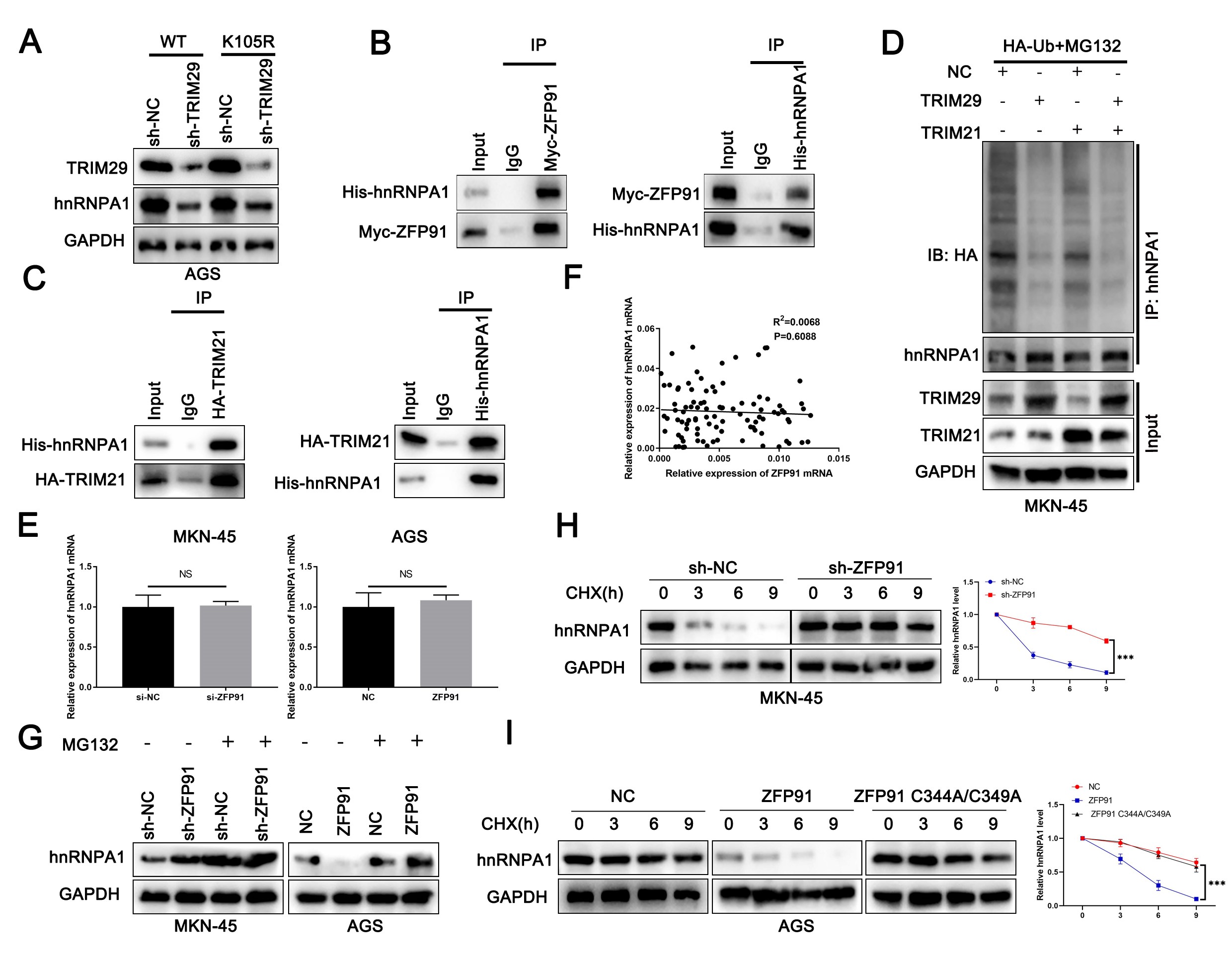 |
| --- |
| **Figure S4. TRIM29 stabilizes hnRNPA1 by preventing ZFP91-mediated K48 ubiquitination. A,** GC cells were transfected with wild-type ubiquitin (HA-Ub) and either wild-type His-hnRNPA1 or the His-hnRNPA1-K105R mutant, and cultured for 72 hours in medium containing control shRNA or shRNA targeting TRIM29. After that, cell lysates were harvested and subjected to western blotting for the analysis of hnRNPA1 and TRIM29 protein levels. **B,** immunoprecipitation were carried out utilizing antibodies specific to Myc-ZFP91 and His-hnRNPA1 to confirm their binding. **C,** immunoprecipitation were carried out utilizing antibodies specific to HA-TRIM21 and His-hnRNPA1 to confirm their binding. **D,** Cells were transfected with either the control vector plasmid or a plasmid expressing TRIM29 or TRIM21, along with HA-Ub. Following transfection, cell lysates were prepared and subjected to IP using anti-hnRNPA1. The precipitated proteins were then analyzed by immunoblotting. **E,** The hnRNPA1 mRNA in GC cell was explored with ZFP91 depletion or overexpression. **F,** The correlation between ZFP91 mRNA and hnRNPA1 mRNA was analyzed based on GC tissues from our center. **G,** Immunoblotting analysis was carried out to evaluate hnRNPA1 expression in GC cells treated with MG132 or not, under ZFP91 silencing conditions or with ZFP91 overexpression. **H,** The stability of hnRNPA1 proteins was assessed in GC cells with ZFP91 depletion, following treatment with cycloheximide (CHX) and subsequent detection at specific time points. **I,** The stability of hnRNPA1 proteins was assessed in GC cells with ZFP91 wild type (WT) or mutant (ZFP91 C344A/C349A) overexpression, following treatment with cycloheximide (CHX) and subsequent detection at specific time points. **P < 0.05, **P < 0.01, ***P < 0.001*. |

| 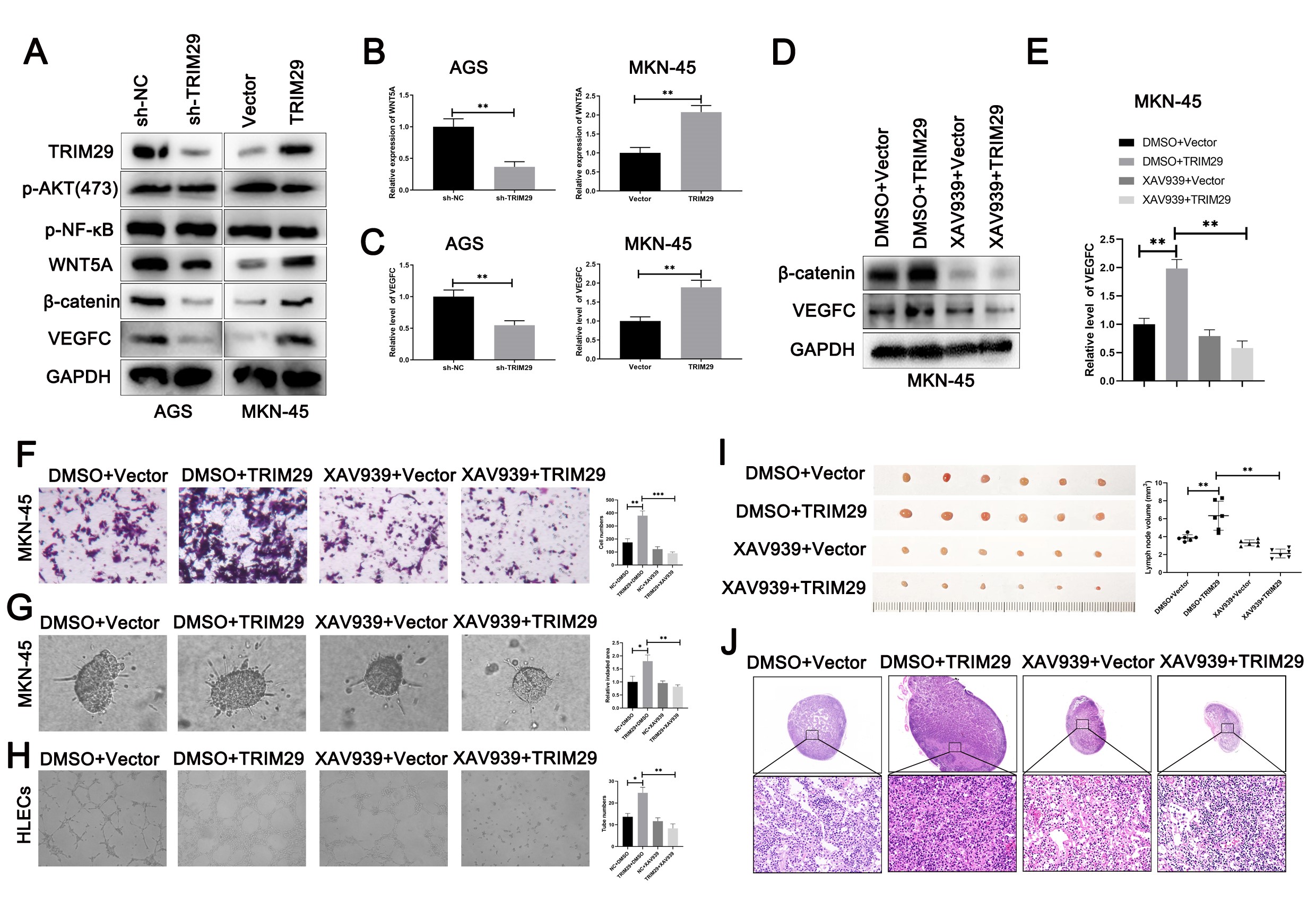 |
| --- |
| **Figure S5. TRIM29-mediated GC malignant behaviors relied on Wnt/β-catenin pathway. A,** Cells were treated with TRIM29 knockdown or overexpression, then the alteration of AKT, NF-κB and wnt/β-catenin signaling was detected through western blot analysis. **B,** The change of WNT5A mRNA level with TRIM29 was probed by qRT-PCR. **C,** Elisa assay was performed to detect released VEGFC level. **D,** TRIM29-overexpressing cells were exposed to XAV-939, an inhibitor of the β-catenin signaling pathway, at a concentration of 5 μM. The subsequent suppression of the β-catenin pathway was confirmed through western blot analysis. To further assess the impact of β-catenin pathway modulation by TRIM29, **E,** Elisa assay detecting VEGFC. Functional assays including **F,** wound healing **G,** 3D invasion, and **H,** tube formation were conducted to evaluate the role of β-catenin signaling pathway on TRIM29 induced GC malignant progression. **I,** **J,** Popliteal LNs metastasis model (XAV-939 at a concentration of 10 mg/kg) was performed to analyze the rescued effect of β-catenin signaling pathway on TRIM29 in vivo. **P < 0.05, **P < 0.01, ***P < 0.001*. |

| 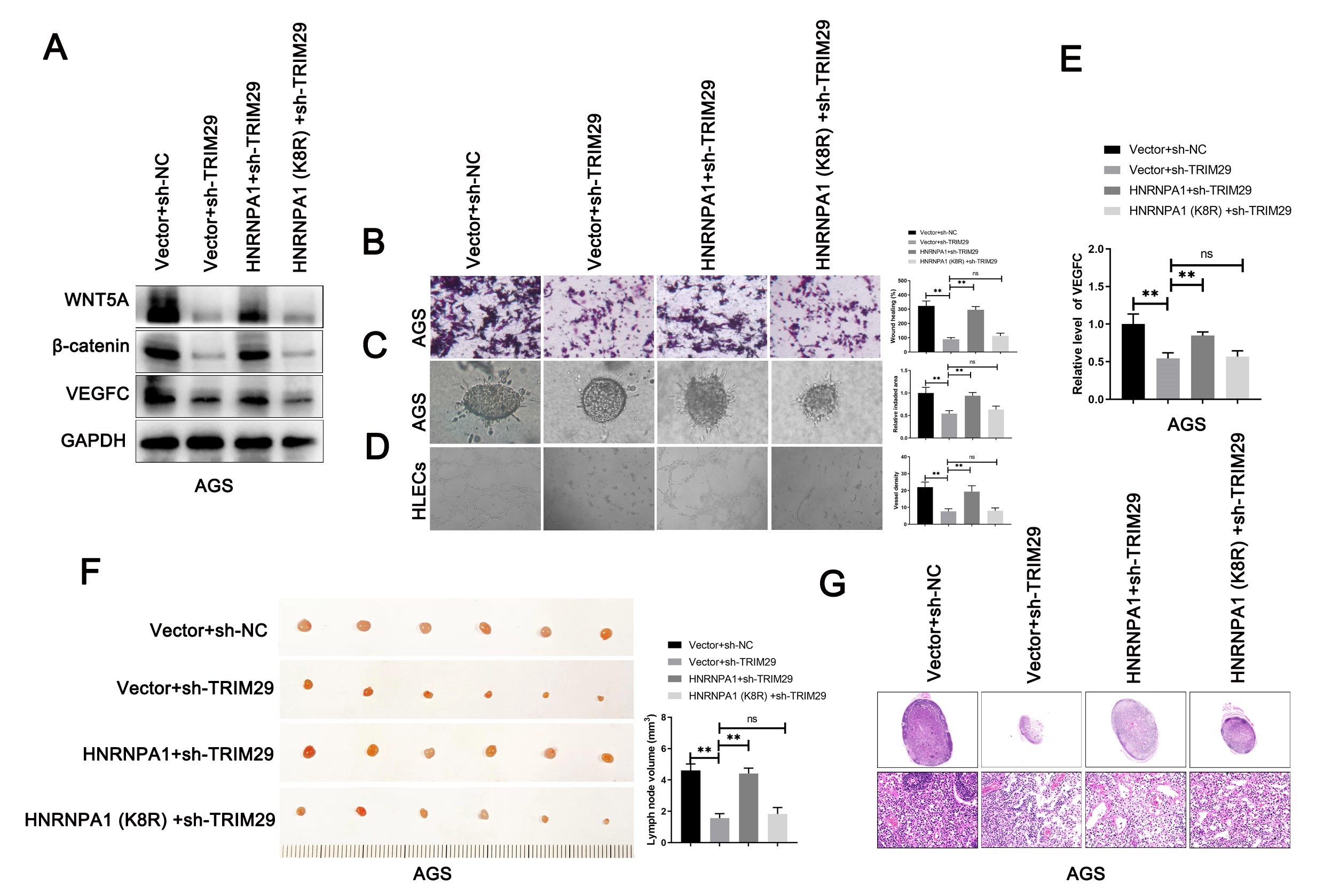 |
| --- |
| **Figure S6. TRIM29-mediated alteration of Wnt/β-catenin pathway was regulated by hnRNPA1. A,** Cells silencing TRIM29 were transfected with hnRNPA1 plasmid, following which the alteration of the wnt/β-catenin signaling was confirmed through western blot analysis. Functional assays, including **B,** wound healing, **C,** 3D invasion, and **D,** tube formation with human liver endothelial cells (HLECs), was conducted to assess the reversed impact of hnRNPA1 on TRIM29 function. **E,** Elisa assay was performed to detect released VEGFC level. **F, G,** Popliteal LNs metastasis model was performed to analyze the rescued effect of hnRNPA1 on TRIM29 in vivo. **P < 0.05, **P < 0.01, ***P < 0.001*. |
