# Supplementary material for: Lactylation-drived TRIM29 induces invasive behavior and lymph node metastasis in gastric cancer via hnRNPA1-mediated Wnt/β-catenin pathway
Source: Cell Death Dis. 2026 Feb 13;17(1):222. doi: 10.1038/s41419-026-08468-9 (PMC12921344; doi:10.1038/s41419-026-08468-9)
Supplement: Supplementary file 2 — Supplemental Material --WB original [file 41419_2026_8468_MOESM2_ESM.pdf]

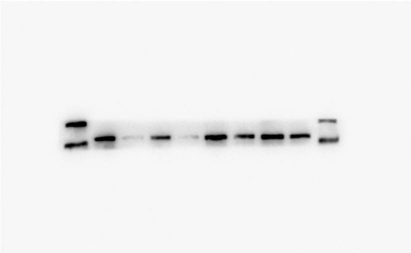	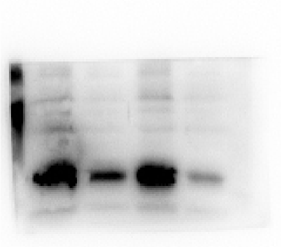	TRIM21
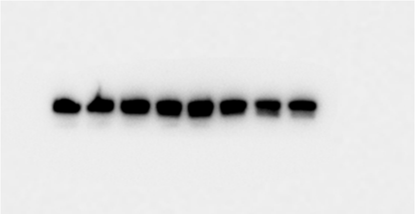	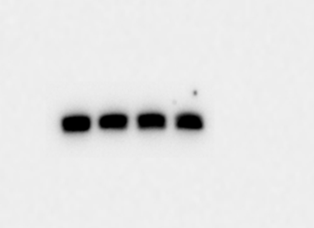	GAPDH
Figure1 E		

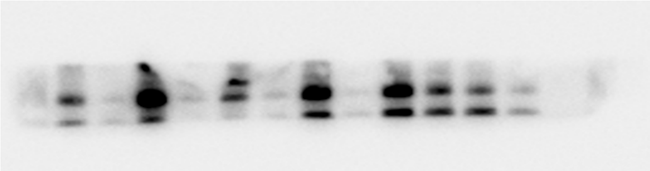	TRIM29
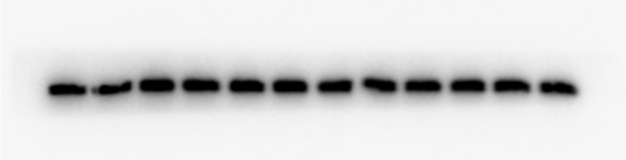	GAPDH
Figure1 G	

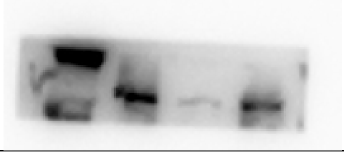	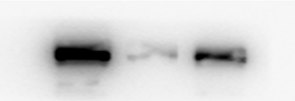
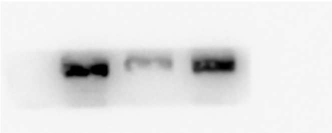	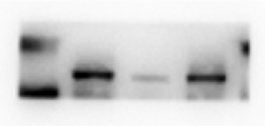
Figure4 B	

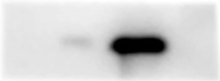	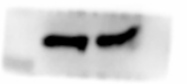
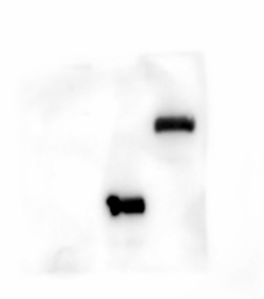	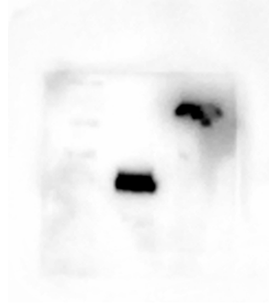
GST pull down	Input
Figure4 C	

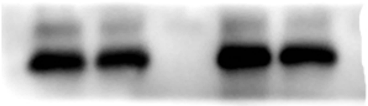	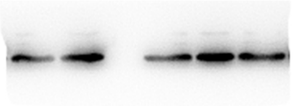
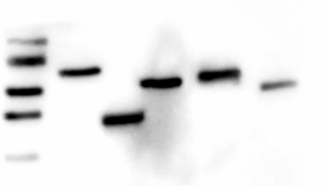	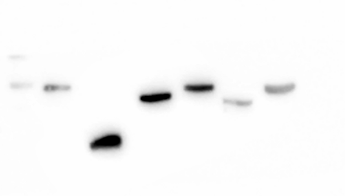
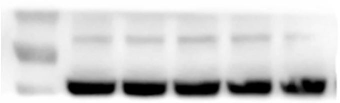	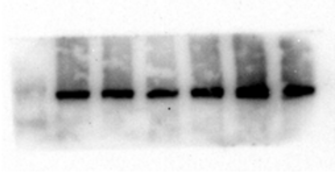
Figure4 D	

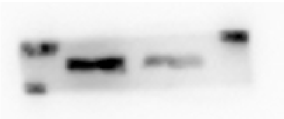	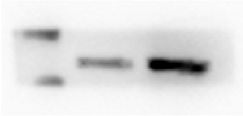	TRIM29
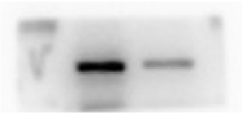	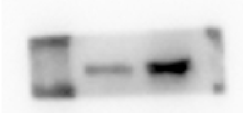	hnRNPA1
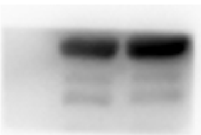	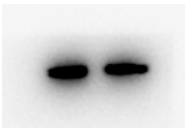	GAPDH

Figure4 E		
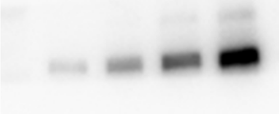	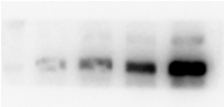	TRIM29
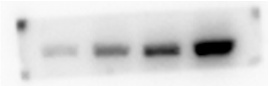	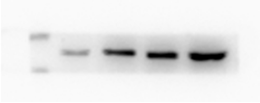	hnRNPA1
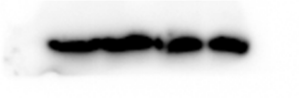	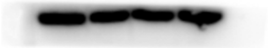	GAPDH
Figure4 F		

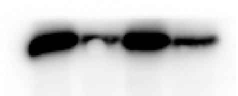	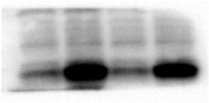	TRIM29
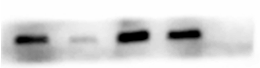	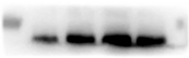	hnRNPA1
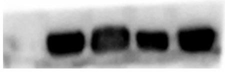	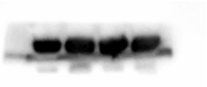	GAPDH
Figure4 G		

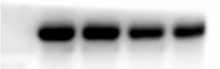	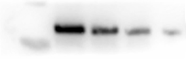	hnRNPA1
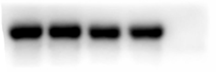	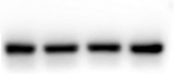	GAPDH
Figure4 H		

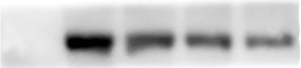	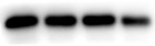	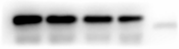	hnRNPA1
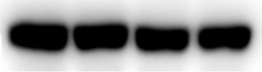	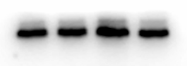	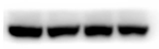	GAPDH
Figure4 I			

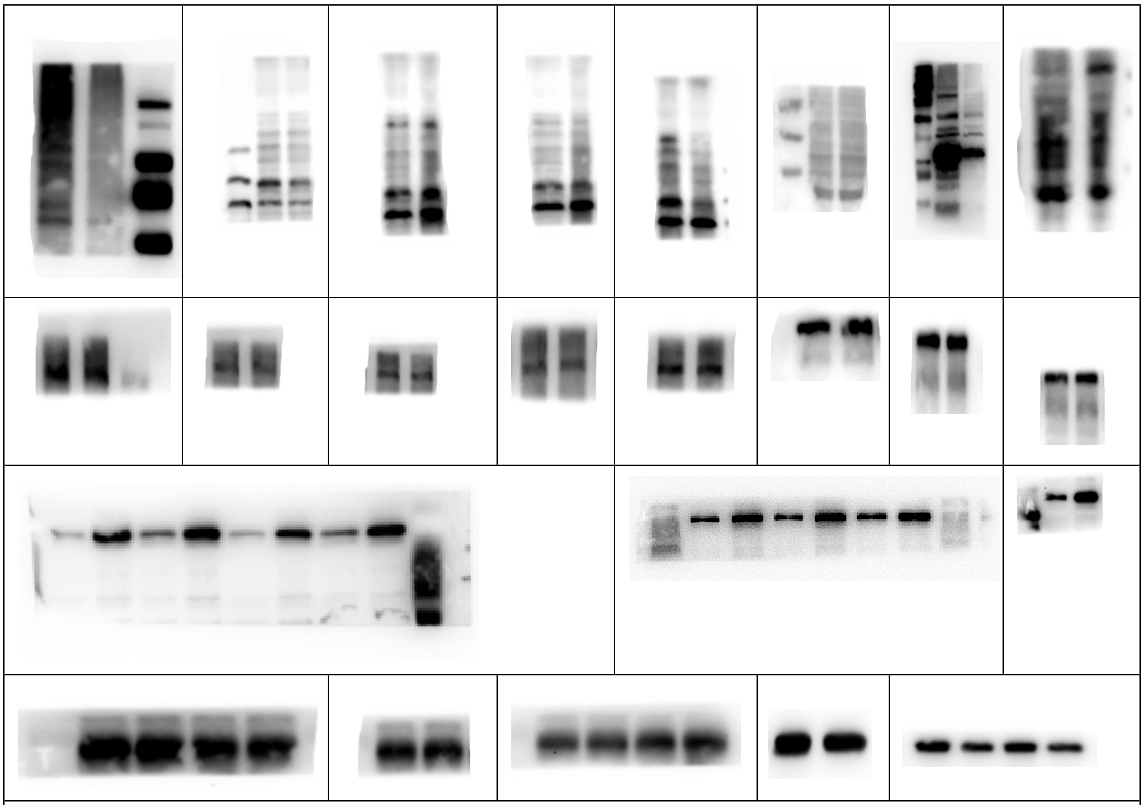

Figure5 A

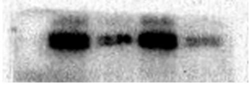	TRIM29
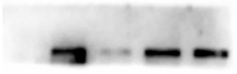	hnRNPA 1
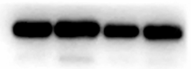	GAPDH

Figure5 B

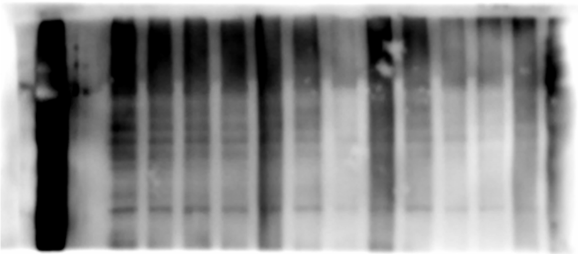	IB：HA
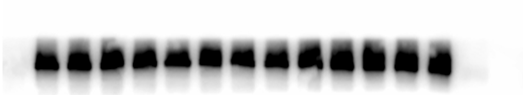	His
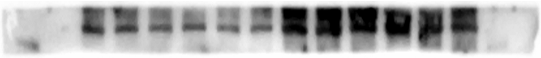	TRIM29
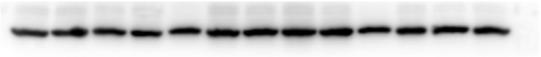	GAPDH
Figure5 C	

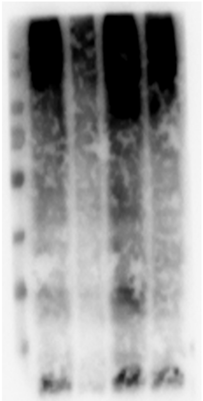	HA
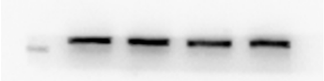	hnRNPA1
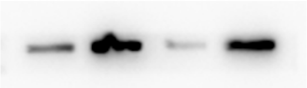	TRIM29
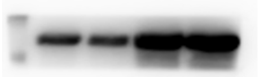	ZEF91
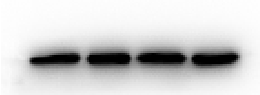	GAPDH
Figure5 E	

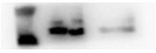	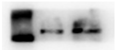	ZEF91
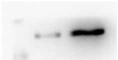	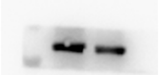	hnRNPA1

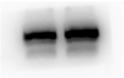	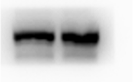	TRIM29
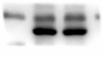	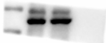	GAPDH

Figure5 F

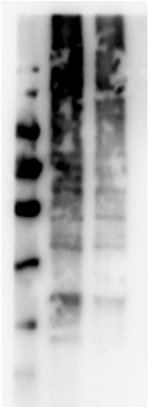	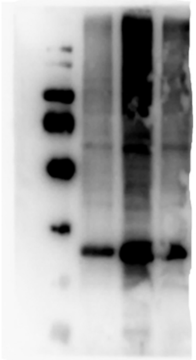	IB：HA
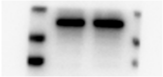	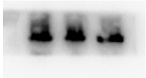	hnRNPA1
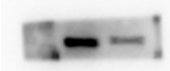	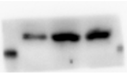	ZFP91
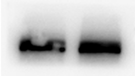	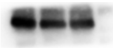	GAPDH

Figure5 G

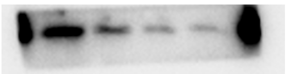	ZFP91
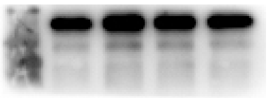	hnRNPA1
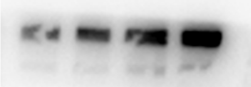	TRIM29
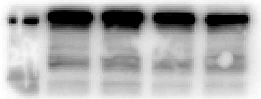	ZFP91
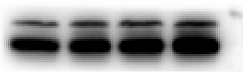	hnRNPA1
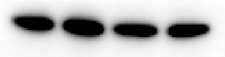	GAPDH

Figure5 H

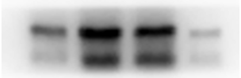	ZFP91
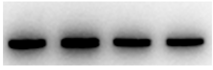	hnRNPA1
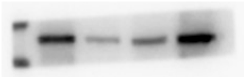	TRIM29
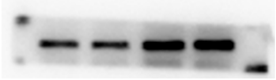	ZFP91
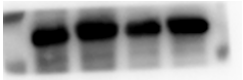	GAPDH

Figure5 I

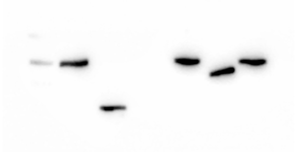	His
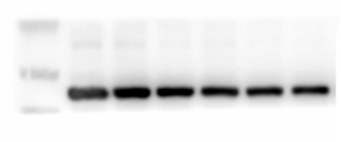	Myc:IP
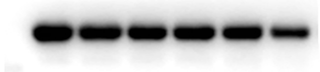	Myc:input

Figure5 J

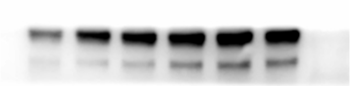	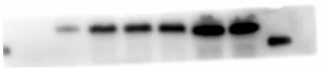	TRIM29
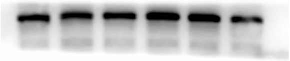	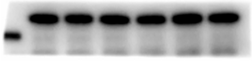	GAPDH

Figure6 B

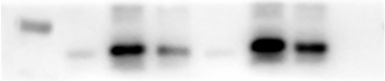	TRIM29
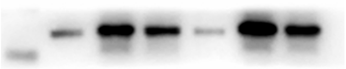	Pan-Kla
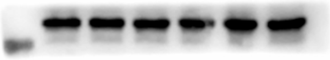	Total H3

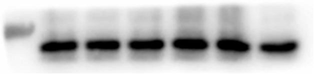	GAPDH
Figure6 C	

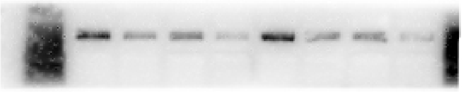	Pan Kla
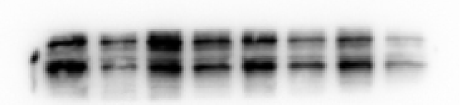	H3k9la
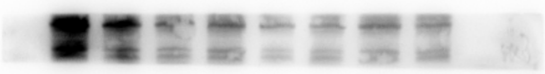	H3k18la
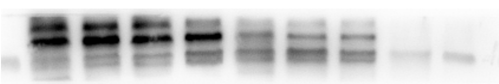	H4k5la
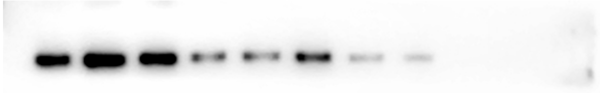	H4k8la
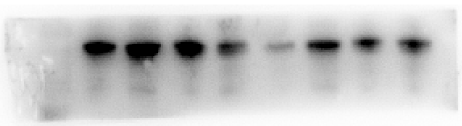	H4k12la
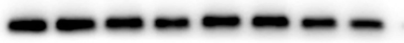	H3
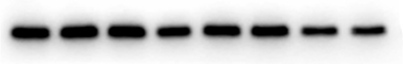	H4
Figure6 E	

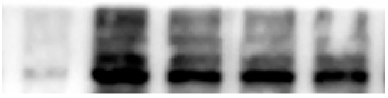	TRIM29
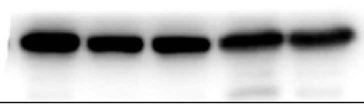	GAPDH
Supplemental Figure 1 D	

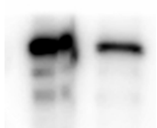	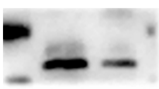	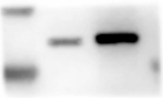	TRIM29

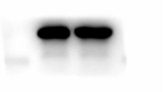	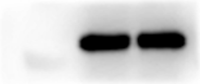	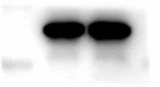	GAPDH
Supplemental Figure 2B			

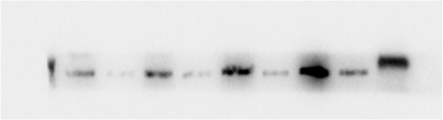	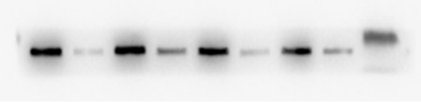	hnRNPA1
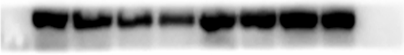	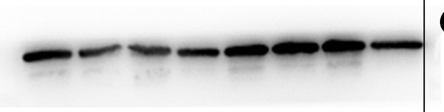	GAPDH
Supplemental Figure 3B		

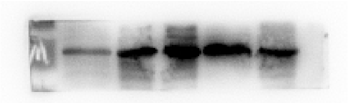	hnRNPA1
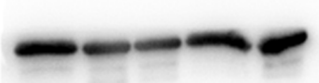	GAPDH
Supplemental Figure 3D	

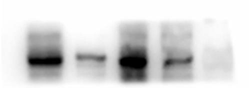	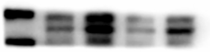	TRIM29
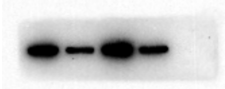	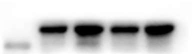	hnRNPA1
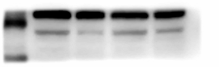	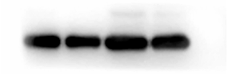	GAPDH

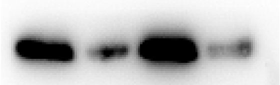	TRIM29
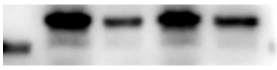	hnRNPA1
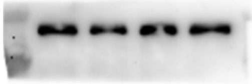	GAPDH
Supplemental Figure 4A	

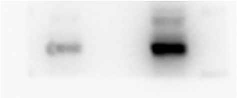	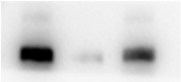
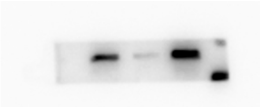	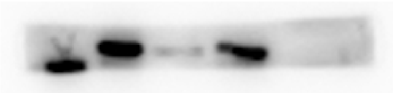
Supplemental Figure 4B	

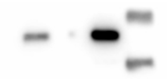	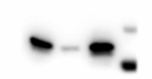
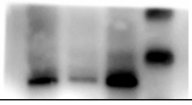	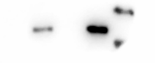
Supplemental Figure 4C	

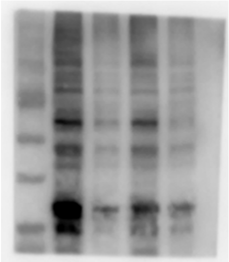	IB:HA
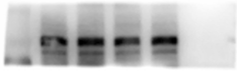	hnRNPA1
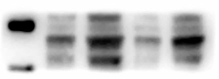	TRIM29
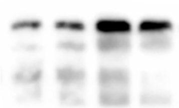	TRIM21
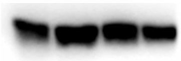	GAPDH
Supplemental Figure 4D	

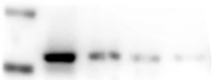	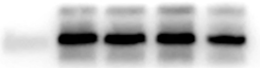	hnRNPA1
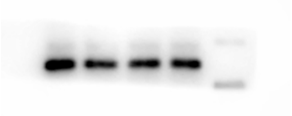	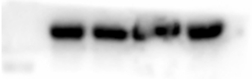	GAPDH
Supplemental Figure 4H		

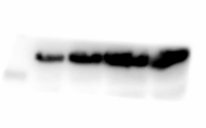	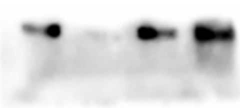	hnRNPA1
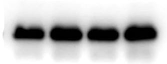	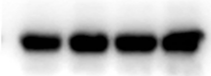	GAPDH
Supplemental Figure 4G		

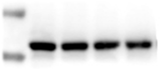		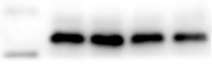	hnRNPA<br>1
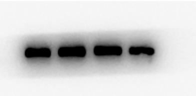	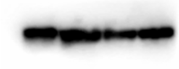	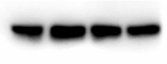	GAPDH
Supplemental Figure 4I			

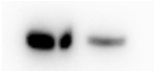	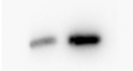	TRIM29
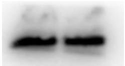	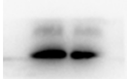	P-AKT
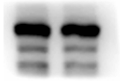	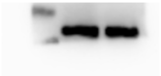	P-nf-kb
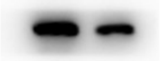	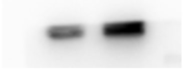	WNT5A
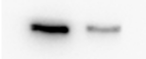	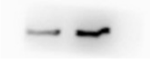	β -catenin
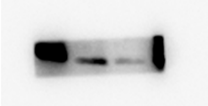	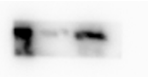	VEGFC
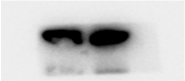	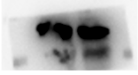	GAPDH

Supplemental Figure 5A

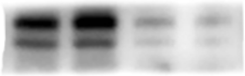	β -catenin
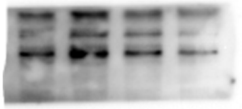	VEGFC
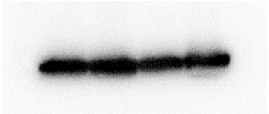	GAPDH
Supplemental Figure 5D	

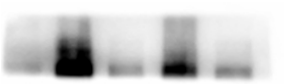	WNT5A
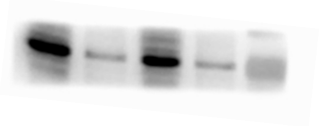	β -catenin
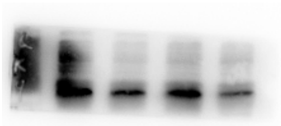	VEGFC
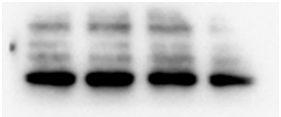	GAPDH
Supplemental Figure 6A	
